# Effect of Cation Symmetry on the Long-Range Ordering in Ionic Liquid Films

**DOI:** 10.1021/acs.langmuir.5c03333

**Published:** 2025-11-14

**Authors:** Colleen B. Lasar, Andrew Horvath, Michael B. Van Den Top, Spyridon Koutsoukos, Tom Welton, Scott K. Shaw

**Affiliations:** † Department of Chemistry, 4083 The University of Iowa, Iowa City, Iowa 52242, United States; ‡ Department of Chemistry, 4615 Imperial College London, London SW7 2AZ, U.K.

## Abstract

This work investigates the role of ionic liquid (IL) ion (a)­symmetry in promoting ordered structures within liquid films by studying a series of six alkylimidazolium cation isomers of varying symmetry paired with a bis­(trifluoromethylsulfonyl)­imide anion. The cation symmetry is varied by systematic variations in the alkyl tail lengths on either side of the imidazolium ring. IL films are extruded on a silver substrate using an in situ dynamic wetting apparatus and allowed to thin under shearing force due to gravity. Film thicknesses are monitored via spectroscopic ellipsometry. Infrared reflection absorption spectroscopy (IRRAS) with p-polarized light is used to analyze changes in dipole moments with vector components perpendicular to the substrate and thus report changes to molecular orientations and local chemical environments. Multiple vibrational modes are monitored at varying film thicknesses to deliver chemical insight into the evolving net molecular orientation within the IL films. Specifically, average molecular orientations are tracked by monitoring intensity and energy shifts of vibrational modes, including the S–N–S ν_as_ (∼1054 cm^–1^), SO_2_ ν_ss_ (∼1137 cm^–1^), and SO_2_ ν_as_ (∼1330 cm^–1^) stretches. This provides a unique ability to determine the extent of ordering in the film. As IRRAS probes the entire film, when changes to the spectral profile cease and only a uniform decrease in absorbance is observed, only the ordered domains of the film remain on the substrate; thus, the film’s thickness is equal to the extent of ordering in the film. Ultimately, for the six alkylimidazolium IL isomers examined here, the extent of molecular ordering in the films increases with increasing asymmetry of the cation. IL ordered domains extend to 0.4 ± 0.2 μm for the most symmetric systems and to 0.7 ± 0.2 μm for the most asymmetric systems.

## Graphical abstract

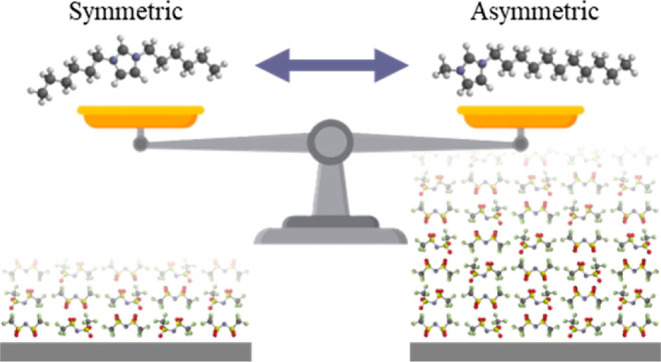

## Introduction

Ionic liquids (ILs) are composed entirely of ions when pure and are liquid at temperatures below 100 °C. When large asymmetric molecular ions are used, melting points, even below room temperature, can be achieved. The discovery of ILs is often credited to Paul Walden in 1914 for his report on ethylammonium nitrate’s (EAN) unique low-melting, good conductivity, and other unique physicochemical properties.^1–3^ However, earlier reports on what we classify today as ILs had already been published, such as those by Prafulla Chandra Rây on EAN in 1911 and by Carl Schall on several low melting salts in 1908.^4,5^ Since then, ILs have gained significant attention from scientists, escalating in the late 1980s, due to their unique physicochemical properties, which make them advantageous for a variety of practical applications.^2,3,6–12^ Many of these applications involve physical or chemical processes that occur at or near interfaces, e.g., in tribological or energy applications. IL properties can be tuned based on the molecular size, symmetry, functional groups, and charge distribution on the cations and anions. Therefore, gaining a fuller understanding of how changing ILs’ molecular structure affects their behavior at the interface is important for optimizing their application.

It is well known that materials behave differently at or near interfaces, compared to the adjacent isotropic bulk phases. These different behaviors are due to molecular interactions, confinement effects, and changes in mobility, often resulting in ordered structures.^13–17^ The fluid interfacial behaviors often extend a few angstroms or nanometers from the adjacent surface and can be enhanced or promoted by temperature changes, electromagnetic fields, self-assembly, and shear. However, previous studies of ILs near solid surfaces, including those from this group, have shown extended ordering of molecules from tens of nanometers to a few micrometers.^18–23^ These studies also show that varying the cation alkyl chain length, and thus the molecular size, can have an effect on interfacial ordering. Surface force apparatus (SFA) and atomic force microscopy (AFM) studies by Gebbie et al. reported that short-chain 1-alkyl-3-methylimidazolium bis­(trifluoromethylsulfonyl)­imide ([C_*n*_C_1_im]­[NTf_2_], *n* = 2, 3, 4) and 1-butyl-1-methylpyrrolidinium bis­(trifluoromethylsulfonyl)­imide ([C_4_C_1_Pyrr]­[NTf_2_]) showed long-range interfacial ordering of up to 10 nm.^19^ Studies by Hossain et al., who were the first to report that ILs display a piezoelectric effect, reported an induced charge density gradient over multiple tens of micrometers.^24,25^ The magnitude of this piezoelectric response was found to be ion structure-dependent, where [C_*n*_C_1_im]­[NTf_2_] (*n* = 4, 6, and 8) exhibited larger piezoelectric coefficients compared to 1-alkyl-3-methylimidazolium tetrafluoroborate [(C_*n*_C_1_im]­[BF_4_], *n* = 4, 8) and 1-alkyl-1-methylpyrrolidinium bis­(trifluoromethylsulfonyl)­imide ([C_*n*_C_1_Pyrr]­[NTf_2_], *n* = 4, 6, 8, 10) ILs. However, interestingly, they report there was little to no change in the piezoelectric response between similar ILs of varying alkyl chain length. This suggests local anisotropic organization and interfacial structuring in ILs can mimic the asymmetry required for piezoelectricity and that while varying the ion constituents affects the strength at which mechanical energy is converted into electrical energy and vice versa, varying only the alkyl chain length does not. Nevertheless, many factors play a role in piezoelectricity beyond structural asymmetry such as chirality and molecular dynamics; therefore, a lack of change in the piezoelectric response does not directly indicate there was no change in the extent of interfacial ordering with a change in alkyl chain length. Thus, further investigation into solely the effect of the alkyl chain length on interfacial ordering via other analytical techniques is needed.

A spectroscopic study by Anaredy et al. showed long-range ordering or locally preferred orientations in thin films of imidazolium-, pyrrolidinium-, or ammonium-based ILs can extend as far as ∼2 μm when paired with [NTf_2_]^−^ anions, reporting a linear correlation of time required to form ordered structures, or maturation time, with the IL viscosity.^20^ The driving forces for this ordering were unclear but suggested to be interfacial, intermolecular, or shear forces within the liquid. They also report that while the maturation time was significantly affected, the resulting film ordering process was nearly identical on gold, silver, and –OH-terminated self-assembled monolayer-modified silver substrates, concluding that the reorientation process is controlled by the properties of the fluid itself and independent of substrate interactions. A following study of triflate-based ILs paired with 1-butyl-3-methylimidazolium ([C_4_C_1_im]^+^) or diethylmethylammonium ([N_122_]^+^) cations displayed ordering of ∼800 nm, despite [C_4_C_1_im]­[OTf] having twice the viscosity of the analogous [NTf_2_]^−^ system, suggesting an important role of the anion in driving the ordering process.^21^ A study of ILs of the same [C_4_C_1_Pyrr]^+^ cation paired with linear or tetrahedral cyano-based anions had ordered films extending to 0.4 and 1.1 μm, respectively, despite similar viscosities.^22^

A more recent study of quasi-spherical IL ions, tetraoctylphosphonium tetracyanoborate ([P_8888_]­[B­(CN)_4_]) and tetra­(propoxymethyl)­phosphonium tetracyanoborate ([P_(3O1),4_]­[B­(CN)_4_]), showed that these liquids did not form any detectably ordered domains. Instead, these ILs showed only minor changes in the spectroscopic profiles assigned to intramolecular configuration changes of the propoxymethyl chain, when compared to isotropic bulk phases.^26^ This is attributed to a lack of preferred orientation due to the spherical nature of the molecular ions, despite the presence of the same intermolecular electrostatic and shear forces as in the previous IL studies mentioned above. Together, these studies suggest that while viscosity impacts the maturation rates of IL films, the ion symmetry plays a fundamental role in driving the IL films’ long-range ordering.

Our present study reports the effects of systematically varying cation (a)­symmetry on IL ordering by modifying the dividing position of an imidazolium ring along a 12-carbon alkyl chain on the extent of ordering within the IL film. While varying cation symmetry does intrinsically affect the alkyl chain lengths, investigating isomers eliminates the added effect of varying molecular size. To the authors’ knowledge, such a symmetry study on IL isomers has not been previously investigated. The extent of ordering will be determined using two surface-sensitive techniques, spectroscopic ellipsometry and p-polarized infrared reflection absorption spectroscopy (IRRAS) equipped with our group’s unique dynamic wetting apparatus, to monitor film thicknesses and changes to molecular orientations, respectively. This allows us the unique ability to prepare fresh films and monitor structural changes over time in situ. This work used silver substrates as they provide an excellent reflective surface for IRRAS, and previous works showed the film maturation process was nearly identical to that of other reflective substrates. During IRRAS, the p-polarized light oscillates parallel to the plane of incidence and perpendicular to the reflective substrate, permitting us to observe only the vibrational modes that have dipole vector contributions that are perpendicular to the substrate. As the molecules reorient and create ordered structures within the film, those changes are visible within the spectral profile as center frequency shifts and intensity ratios between modes. Herein, we monitored the reorientation process for all six IL isomers observed and demonstrated that increasing the asymmetry of the cation will lead to an increased long-range ordering.

## Experimental Section

### Materials

#### Ionic Liquids

The six ILs, 1-methyl-3-undecylimidazolium bis­(trifluoromethanesulfonyl)­imide ([C_1_C_11_im]­[NTf_2_]), 1-decyl-3-ethylimidazolium bis­(trifluoromethanesulfonyl)­imide ([C_2_C_10_im]­[NTf_2_]), 1-nonyl-3-propylimidazolium bis­(trifluoromethanesulfonyl)­imide ([C_3_C_9_im]­[NTf_2_]), 1-butyl-3-octylimidazolium bis­(trifluoromethanesulfonyl)­imide ([C_4_C_8_im]­[NTf_2_]), 1-heptyl-3-pentylimidazolium bis­(trifluoromethanesulfonyl)­imide ([C_5_C_7_im]­[NTf_2_]), and 1,3-dihexylimidazolium bis­(trifluoromethanesulfonyl)­imide ([C_6_C_6_im]­[NTf_2_]), were synthesized and characterized according to previously published procedures.^27,28^

ILs were stored in a vacuum desiccator, and the water content in the ILs was quantified before use. After experimentation, ILs were recovered by rinsing the IL films from the apparatus with acetone (Fisher Scientific, ACS grade) and collecting the acetone rinsate in a clean glass vial. Acetone was removed by evaporation at room temperature, followed by drying on a Schlenk line (<1 Torr) with stirring and gentle heating (<40 °C) for at least 72 h. These “recovered” ILs were stored in a vacuum desiccator in separate vials from the fresh ILs until needed. Purity of the recovered liquids was monitored with Nuclear Magnetic Resonance (NMR). The ^1^H NMR spectra of the recovered liquids match those of the as-synthesized liquids (see Figure S1).

#### Substrates

Polycrystalline silver disks, 14 mm in diameter, were used as solid, reflective substrates for acquiring reflection-based spectroscopic measurements. The silver disks were milled from 99.999% pure silver rods (ESPI Metals, Portland, OR) and mechanically polished until a mirror finish was achieved using 600 then 100 grit sandpaper (3M), followed by 9.5, 3.0, 1.0, and then 0.3 μm aluminum oxide powder (Buehler) on the clean corresponding Buehler felt or synthetic polishing pads. The disks were then chemically polished using concentrated H_2_SO_4_ (ACS grade, Sigma-Aldrich), HClO_4_ (70%, BDH), 4.0 M chromic acid (CrO_3_, 99.9%, Alfa Aesar), 0.6 M HCl (ACS grade, BDH), and NH_4_OH (28.0–30.0%, Sigma-Aldrich), to achieve a clean, smooth, and reflective surface.^29^ H_2_SO_4_, HClO_4_, and NH_4_OH were used as received. All other solutions were prepared with water from a Milli-Q Advantage A10 System (Millipore Corp, 18.2 MΩ/cm resistivity, TOC <4 ppb). After polishing, the silver substrate was dried with N_2_ gas (Praxair, 99.995%), immediately affixed to the dynamic wetting cell (detailed below), and placed under a N_2_ atmosphere for experimentation. After experimentation, the substrate was stored in Milli-Q water until repolishing. The surface cleanliness and roughness were monitored via *n* and *k* values from ellipsometry measurements and matched constant values for the bare metal.^30,31^

#### Water Content

Water contents were quantified using an 831 Karl Fischer (KF) Coulometer with a generator electrode with a diaphragm (Metrohm, Switzerland). The inner and outer KF cells were filled with Fluka HYRANAL-Coulomat CG (Sigma-Aldrich, Lot# ZBF044DV) catholyte and HYRANAL-Coulomat AG (Honeywell, Lot# L2510) anolyte, respectively, and the anolyte solution was briskly stirred during measurements to ensure solution homogeneity. Instrument accuracy was verified for quality control using HYDRANAL-Water Standard 1.0 (Sigma-Aldrich, Fluka, Lot#SZBF2500 V). An IL sample size of 0.36 ± 0.05 g (∼200 μL) was used for all measurements. Each IL was found to contain <50 ppm water prior to sample storage and further experimentation (Table 1). Due to limited materials and low initial water content, KF was not tested again after drying on the Schlenk line; however, water content was monitored via associated vibrational modes during IRRAS measurements.

**Table 1:** Physicochemical Properties of [C_*n*_C_12–*n*_im]­[NTf_2_] at Room Temperature^a^

ionic liquid	water content, ppm	density at RT, g/mL	viscosity at 25 °C, cP	surface tension at RT, mN/m	cation diffusion coefficient at 25 °C, D_+_ (10^–12^ m^2^ s^–1^)	anion diffusion coefficient at 25 °C, D_–_ (10^–12^ m^2^ s^–1^)
[C_1_C_11_im][NTf_2_]	26.4	1.15 ± 0.04	124.7 ± 0.3	25 ± 2	7.7 ± 0.1	9.2 ± 0.1
[C_2_C_10_im][NTf_2_]	13.7	1.05 ± 0.03	137.9 ± 0.4	22.5 ± 0.6	10.1 ± 0.1	8.4 ± 0.1
[C_3_C_9_im] [NTf_2_]	49.3	1.18 ± 0.04	103.5 ± 0.3	25.6 ± 0.6	9.3 ± 0.1	9.5 ± 0.1
[C_4_C_8_im] [NTf_2_]	26.7	1.00 ± 0.03	103.4 ± 0.3	22.8 ± 0.5	9.4 ± 0.1	9.5 ± 0.1
[C_5_C_7_im] [NTf_2_]	12.4	1.18 ± 0.04	104.9 ± 0.3	25.5 ± 0.6	9.1 ± 0.1	10 ± 0.1
[C_6_C_6_im] [NTf_2_]	29.1	1.14 ± 0.04	89.2 ± 0.2	25.4 ± 0.4	8.7 ± 0.1	8.9 ± 0.1

a Errors are the standard deviation of *n* ≥ 3 measurements when listed.

#### Density

The density of the six IL samples was determined by using the direct density method. A VWR Ergonomic High Performance micropipette (100–1000 μL, ±1.6 μL error) was used to dispense 100 μL of the sample, and the mass was measured using a Mettler Toledo NewClassic MF analytical balance (model M51015DU, ±0.08 mg error).

Density was also measured in an Anton Paar DMA 5000 M density meter. The density meter is based on a U-based vibrating tube, which is excited electronically to oscillate at its characteristic frequency. The frequency changes depend on the density of the filled sample; therefore, the true density is calculated by measuring that frequency. The precision of the measurements was determined by performing standard measurements in air and in MQ water and was found to be 0.000050 g mL^–1^ (for the density) and 0.001 °C (for the temperature). Detailed methodology can be found in previous publications.^28^

#### Viscosity

Viscosity measurements were acquired on a DV2T-LV Cone/Plate Viscometer (Brookfield) equipped with a CPA-40Z spindle and TC-550 Temperature Control Circulating Bath, which recirculates a water/glycol solution (Brookfield, TC-Fluid 2) through the CPA-44Y cup. Standards were collected for method quality control using Certified Viscosity Standard Number B29/PAO oil (Brookfield Engineering Laboratories Inc., Middleboro, MA, CAS# 68037–01–4, Lot No. 17101, 29.50 cP at 25.00 °C), which had a viscosity of 29.60 ± 0.1 cP at 25.0 °C. All measurements were acquired at 25 °C in triplicate on three separate 500 μL aliquot samples each (*n* = 9).

Viscosity was also measured using a LOVIS 2000 ME microviscometer, based on Hoeppler’s falling ball principle. The accuracy of the module is 0.05% (for viscosity) and 0.02 °C (for temperature). Detailed methodology can be found in previous publications.^28^

#### Self-Diffusion Coefficient

Pulsed-field gradient stimulated echo (PFGSTE) experiments were performed at 297 K using a Bruker Avance III HD 500 NMR spectrometer, equipped with a 5 mm BBO SmartProbe. The samples were dried and degassed before each measurement. The complete methodology can be found in detail in previous publications.^32,33^

#### Surface Tension

Surface tension measurements were acquired via pendant drop analysis using a goniometer (Ramé-Hart model 100) coupled with 6–60× magnification lenses and a high-resolution CMOS camera (Thor Laboratories) under ambient conditions. A 1000 μL VWR Ergonomic High-Performance micropipet with a 1.16 ± 0.06 mm o.d. pipet tip was used to dispense droplets while a video was recorded. ImageJ software with the drop analysis plugin was used to analyze and determine surface tension values from still frames (*n* ≥ 9) captured of the IL droplets while the pipet tip was still in the droplet just before the droplet released.

#### Dynamic Wetting Apparatus

IL films were prepared and analyzed using a custom-made dynamic wetting apparatus and enclosure described previously.^20^ A polished silver substrate attached to a brass rod is swiftly inserted into a poly­(tetrafluoroethylene) cell and made to rotate with a small DC motor and 10683:1 gearbox (Micromoto, Clearwater, FL). The cell was sealed and purged with N_2_ gas (Praxair, 99.995%) and held at a slight positive pressure (∼2–4 psi) to maintain a controlled gas environment during experiments. Spectroscopic measurements were conducted in a reflection geometry, allowed by two UV-grade 1 mm thick × 25 mm diameter CaF_2_ optical windows (Crystran, UK) placed on either side of the silver substrate. The IL was introduced into the cell via a glass capillary (1 mm i.d.) positioned with a ∼1 mm gap between the glass capillary and the substrate surface. A small droplet (∼0.05 mL) of IL was dispensed from the capillary and held between the capillary and the substrate due to surface tension and capillary forces. As the silver surface rotated through the droplet, a fluid thin film was extruded onto the substrate surface. The rotation speed was controlled using a custom variable voltage (0–12 V) motor power supply. For this study, a rotational velocity of 60 μm/s (∼0.1 rpm) measured at the perimeter of the silver substrate was used. The same sampling geometry and conditions were used for both ellipsometry and IRRAS measurements.

#### Ellipsometry

Ellipsometry measurements were conducted on an M-2000 V Spectroscopic Ellipsometer with an EC-400 controller (J.A. Woolam Co., Inc.) with an incident angle of 78 ± 3° with respect to the surface normal and spectral window of a wavelength of 350–1000 nm. Before the IL sample was introduced, a background was collected from the freshly polished, bare silver substrate. Then the IL sample was introduced to the rotating silver substrate with a rotational velocity of 60 μm/s, and a film was allowed to form for one hour to ensure uniform film thickness. Rotation was then stopped, and ellipsometry data were collected at 10 min intervals for 10 h. Δ and Ψ values, which represent the phase difference and amplitude ratio of p- and s-polarized components of reflected light, respectively, were used to create fitted models within CompleteEase software, yielding film thickness values. The models (developed in collaboration with J.A. Wollam scientists) included a bare silver substrate layer, an intermix layer (∼50 nm), and a general oscillator layer to account for any absorption by the IL film.

#### FTIR Spectroscopy

FTIR spectra were acquired on a ThermoScientific Nicolet iS50 Advanced Fourier Transform Spectrometer with a liquid-nitrogen-cooled MCT-A detector. Transmission spectra were collected of a 4 μL aliquot of IL sample that was pressed between two calcium fluoride optical windows (CaF_2,_ 15 mm dia., Crystran, UK). Reported spectra were averaged over 32 scans recorded at a 4 cm^–1^ resolution. Unless otherwise stated, transmission IR data are representative of triplicate spectra collected for each of three separate aliquots, for *n* = 9.

#### IRRAS

Infrared reflection absorption spectroscopy (IRRAS) measurements were acquired on the same iS50 spectrometer equipped with an external tabletop optical mount (TOM box, Newport) purged with CO_2_-free dry air, a wire grid polarizer, an external MCT-A detector, and the same custom dynamic wetting cell set described above. Spectra were averaged over 1000 scans at 4 cm^–1^ resolution with an optical velocity of 1.8988. Background IRRAS measurements were acquired on a freshly polished bare silver substrate prior to the introduction of the IL sample to check for contamination. IL films were created using the same procedure as that for ellipsometry experiments. IRRAS spectra were collected while the silver substrate was rotating, then the rotation was stopped, and additional spectra were acquired as the film thinned for ∼ 10 h. Vibrational absorption mode peak centers (wavenumbers) and associated absorbance values were quantified from the IR spectra using OriginLab Pro software.

#### NMR

NMR data were acquired with a Bruker NEO-500 (Bruker Biospin, Billerica, MA) spectrometer equipped with a 5 mm probe (PABBO BB-1H/D Z-GRD). The spectrometer was controlled with Topspin software (version 4.1.1). The NEO-500 spectrometer was operated at a ^1^H frequency of 500.3 ppm. The temperature of the samples was controlled by a gas stream passing over the sample and heated as necessary to achieve the desired temperature (typically 300 K). The receiver gain was adjusted as appropriate for each spectrum. Recycle delays were 5 s for ^1^H with a 30-degree excitation pulse (15 μs, 16.6 kHz). Chemical shift was set via the solvent resonance to correspond to TMS = 0 (e.g., CD_3_CN was referenced as 1.94 ppm).

#### Differential Scanning Calorimetry

Phase transition temperatures and their associated enthalpies were measured by monitoring heat flow as a function of temperature and time on a Q100 TA Instruments differential scanning calorimeter equipped with a liquid nitrogen cooling system. IL samples (4 μL) were prepared in an aluminum crucible without a lid. The samples were then heated in situ to 120 °C for 30 min to dry off any residual water within the IL. The samples were then cooled to −150 °C at 5 °C/min, held at −150 °C for 1 min or until thermal equilibrium, and then heated at 5 °C/min to 40 °C. Thermograms were analyzed using TRIOS. Glass transitions were determined using the half-height midpoint type, and first-order phase transitions were integrated using linear baselines.

## Results and Discussion

To investigate the influence of cation (a)­symmetry on the spontaneous ordering of ILs in thin films, six aprotic ILs containing isomeric cations ([C_*n*_C_12–*n*_im]^+^, *n* = 1–6), where cation symmetry increases with n, and the same anion ([NTf_2_]^−^) were studied: [C_1_C_11_im]­[NTf_2_], [C_2_C_10_im]­[NTf_2_], [C_3_C_9_im]­[NTf_2_], [C_4_C_8_im]­[NTf_2_], [C_5_C_7_im]­[NTf_2_], and [C_6_C_6_im]­[NTf_2_]. Each cation contains 12 total carbons within the two alkyl chains. The imidazolium ring is displaced from the “center” of the two alkyl tails, moving one carbon along the chain, much like sliding the pull tab along the teeth of a zipper, as illustrated in the molecular structures as seen in Figure 1.

**Figure 1:**
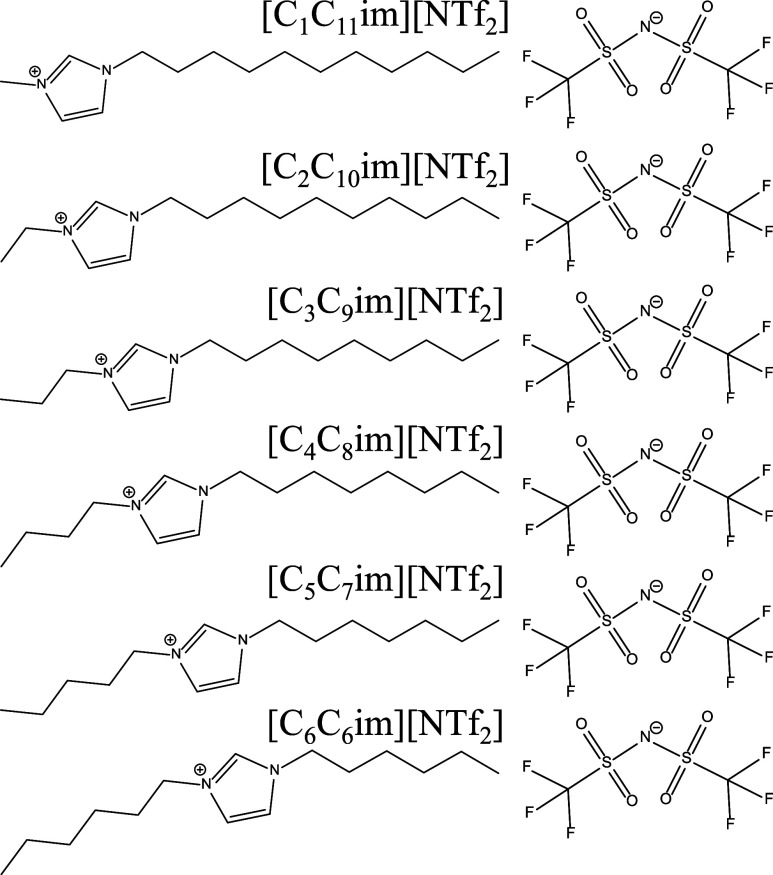
Molecular structures of the six “zipper” ionic liquids used in this study.

Our measured density, viscosity, surface tension, and diffusion coefficient values for each of the six ILs are listed in Table 1. As the six ILs are isomers with the only structural change being the length of the alkyl chains on either side of the imidazolium ring, it was expected that these properties would be similar, except for a slight increase in viscosity with increased asymmetry, as seen in studies with similar ILs.^34,35^ This increase in viscosity with increased asymmetry is attributed to two main factors: the first being increased Coulombic interactions due to the ability of the anion to approach the cation and increased dispersion forces between the long alkyl chains and the related polar and apolar nanodomain formation, as shown in studies involving similar ILs.^36,37^ The influence of these nanodomains were demonstrated in a study by Borah et al., which showed that upon cooling, trihexyltetradecylphosphonium bis­(trifluoromethylsulfonyl)­imide ([P_666,14_]­[NTf_2_]) undergoes a liquid–liquid phase transition in a two-stage process, where the polar charged nanodomains slowdown, or “freeze”, first then the apolar long alkyl chain nanodomains slowdown at lower temperatures (Δ 60 °C).^38^ Thus, [C_6_C_6_im]­[NTf_2_], being the most symmetric, was expected to be the least viscous IL and indeed had the lowest viscosity of 89.2 ± 0.2 cP at 25 °C. The ILs generally increased in viscosity with increased cation asymmetry, with the interesting exception of [C_2_C_10_im]­[NTf_2_], which had the highest viscosity despite not being the most asymmetric. At 137.9 ± 0.4 cP at 25 °C, [C_2_C_10_im]­[NTf_2_] has a viscosity ∼ 13 cP higher than that of our most asymmetric IL in the series, [C_1_C_11_im]­[NTf_2_]. This could be attributed to [C_2_C_10_im]­[NTf_2_] having the slowest anion diffusion coefficient yet also the fastest cation diffusion coefficient of the six ILs. Furthermore, [C_2_C_10_im]­[NTf_2_] has a faster anion than the cation diffusion coefficient, whereas most ILs in this study have faster cation than anion diffusion coefficients.

These physicochemical properties were then used with the Landau–Levich (LLD) model to calculate the theoretical film thickness at a given withdrawal speed for each respective IL. The LLD model is a useful relationship of a Newtonian fluid’s film thickness on a solid substrate with the withdrawal speed of that substrate from a fluid reservoir.^39^ As shown in the equation, the film thickness (*h*, m) is directly proportional to viscosity (η, cP) and withdrawal speed (*U*, m/s) and inversely proportional to surface tension (γ, N/m), density (ρ, kg/m^3^), and acceleration of gravity (*g*, m/s^2^).

$$h=0.299\frac{{\left(\eta U\right)}^{2/3}}{\gamma^{1/6}{\left(\rho g\right)}^{1/2}}$$

Previous studies have demonstrated that some ILs exhibit non-Newtonian behavior and have physicochemical properties that cause greater film thicknesses than predicted by the LLD model.^40–42^ While the degree of accuracy of the LLD model on IL systems is dependent on the degree of intermolecular interaction or non-Newtonian behavior, previous work in this group has shown that the LLD model accurately predicts IL + water mixtures due to the increased hydrogen bonding network. In addition, the LLD model is a reasonable approximation for neat IL film thickness (within ∼ 1 μm at withdrawal speeds <80 μm/s).^30^ This is because the LLD model is based on three key assumptions: the solid substrate surface area is significantly larger than the film thickness, the film thickness is uniform across the surface, and the withdrawal speed of the surface is slow enough that the first molecular layer of the IL in contact with the solid substrate can be considered static, all of which are applicable in this study.

The experimental film thicknesses for each of the six ILs measured using ellipsometry, as described above, are reported in Table 2. The ILs exhibited ∼2–6 μm thicker films than the LLD model predicted, as observed in previous studies.^20–22,30,40,42,43^ This deviation from the model indicates that the six ILs in this study exhibit greater non-Newtonian behavior and suggest more complex intermolecular interactions and dynamic structure than the ILs in the previous studies. Still, experimental film thicknesses qualitatively followed the LLD predicted trends.

**Table 2:** Film Thickness and Maturation Time of ILs in This Study^a^

ionic liquid	LLD model predicted thickness @60 μm/s, μm	measured initial film thickness^b^ @60 μm/s, μm	maturation film thickness (μm)	maturation time (min)
[C_1_C_11_im][NTf_2_]	2.00 ± 0.04	6 ± 4	0.7 ± 0.2	540 ± 10
[C_2_C_10_im][NTf_2_]	2.27 ± 0.04	8 ± 2	1.1 ± 0.9^c^	≥600
[C_3_C_9_im][NTf_2_]	1.73 ± 0.03	3 ± 2	0.6 ± 0.3	360 ± 90
[C_4_C_8_im][NTf_2_]	1.91 ± 0.03	7 ± 4	0.64 ± 0.08	470 ± 70
[C_5_C_7_im][NTf_2_]	1.75 ± 0.03	5 ± 2	0.6 ± 0.3	430 ± 20
[C_6_C_6_im][NTf_2_]	1.60 ± 0.03	4 ± 2	0.4 ± 0.2	570 ± 20

a Error is the standard deviation of *n* ≥ 3 measurements.

b Measured film thicknesses determined from ellipsometry and IRRAS measurements.

c Film thickness at 600 min as maturation occurred outside the experimental window.

After ceasing substrate rotation, the film thickness rapidly decreased within the first two hours before plateauing to thicknesses of ∼0.3–1 μm, as seen in Figure 2. This thinning is due to gravitational and shearing forces. Interestingly, during the first few hours, the films develop a small thickness dependency on the “odd–even” alkyl chain length. This relationship can be observed in Figure S3, where the film thicknesses over time are plotted versus the number of carbons on the shorter alkyl chain. Based on the standard deviation error bars, this “odd–even” effect is not statistically significant; however, it is still an interesting phenomenon to note as this relationship has been previously reported to affect conformation and tilt angles of alkyl-based moieties.^44,45^ At the same time points since ceasing rotation, ILs with an even number of carbons on the alkyl chains have slightly thicker films than those with an odd number of carbons. This is most evident for our most asymmetric cations; the effect becomes muted or negligible beyond *n* > 4. Then, after 50 min of ceasing rotation, this odd–even effect is most prominent and is observed for all ILs in the series. Finally, after 150 min, the IL films behave according to a more viscosity-based film thickness correlation. The effect is minor, yet our results suggest an “odd–even” effect on the rate at which the ILs thin over time, where odd-numbered carbon chains result in faster thinning films than those with even numbers.

**Figure 2:**
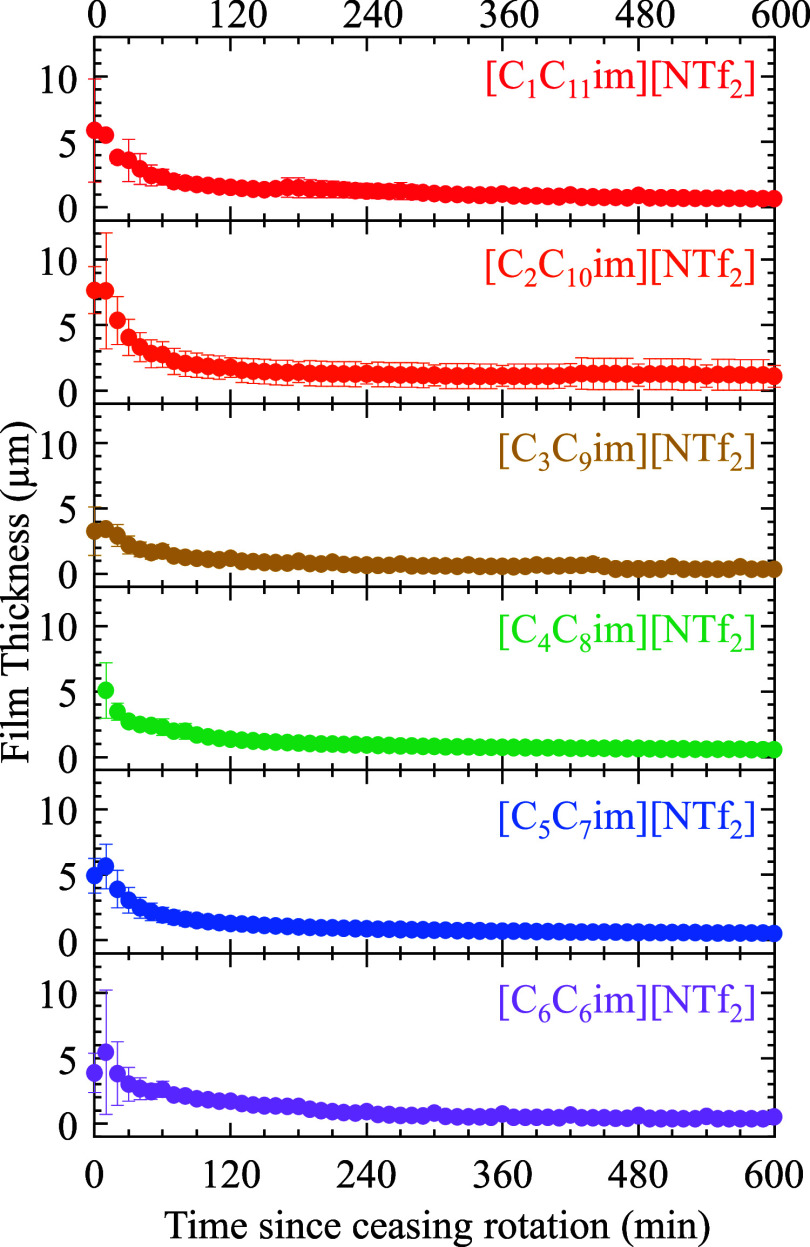
Film thicknesses of the ionic liquid in this study measured by spectroscopic ellipsometry over time after ceasing rotation at 60 μm/s. Error bars represent the standard deviation of *n* ≥ 3 independent trials.

As the IL films became thin, changes in vibrational modes were monitored using IRRAS to confirm and characterize the formation of ordered structures or maturation within the IL films over time. The use of p-polarized infrared light allows us to observe only vibrational modes with a portion of their dipole moment perpendicular to the substrate, and thus, we were able to monitor changes in molecular orientation, as reported previously.^20^ Once rotation of the substrate is stopped, the changes in the infrared profile include shifts in peak absorption energies and relative peak absorbance values, indicative of dipole (molecular) reorientation and changes in intermolecular interactions as ions in the film adopt an increasingly uniform orientation, even as the film thins. Ultimately, the ions in the fluid film reach a steady conformation or maturation point, which is identified by the IR profile changing only by decreasing absorbance intensity. Spectral profiles that illustrate the maturation process are shown in Figure 3. Here, a film of [C_4_C_8_im]­[NTf_2_] is tracked from its initial formation through maturation and subsequent thinning. Specifically, the red trace shows the IR profile while the film is on the rotating substrate (60 μm/s). The “mature” film, highlighted in blue, taken ∼470 min after rotation is stopped, and it shows a near complete loss of intensity for CF_3_ asymmetric stretches (ν_as_) at ∼1226 cm^–1^ and ∼1240 cm^–1^. This reduced intensity is due to the rotation of the associated dipoles being predominantly orthogonal to the incident p-polarized IR beam. In addition, in comparison to fresh films, the spectral profiles of matured films show shifts from isotropic to anisotropic states of the S–N–S ν_as_ stretch, SO_2_ symmetric stretch (ν_ss_), CF_3_ ν_as_, SO_2_ asymmetric in-phase stretch (ν_as_(ip)), and out-of-phase (ν_as_(op)) stretch from 1066 cm^–1^, 1145 cm^–1^, 1165 cm^–1^, 1335 cm^–1^, and 1364 cm^–1^ to 1053 cm^–1^, 1134 cm^–1^, 1177 cm^–1^, 1329 cm^–1^, and 1345 cm^–1^, respectively. Finally, the green trace shows the same “profile” as the blue trace but is characterized by a monotonically lower intensity due to continued thinning of the fluid film after maturation. This denotes that further molecular reorientation does not occur, and the film’s ordered structure is consistent after maturation is achieved. Figure S4 shows the same series of IRRAS spectra at hourly intervals in a plot offset vertically for the sake of clarity.

**Figure 3:**
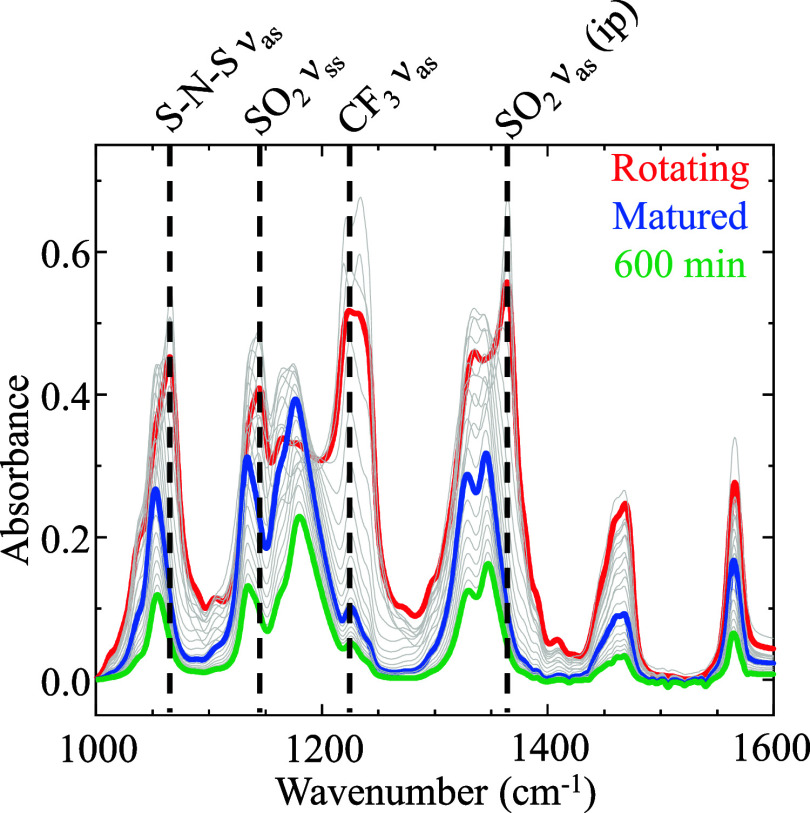
IRRAS spectra displaying the spectral changes as a [C_4_C_8_im]­[NTf_2_] film matures after rotation of the substrate has stopped. Highlighted in red is the film as it is rotating at 60 μm/s, blue is when the film has fully matured, and green is after 10 h have elapsed after ceasing rotation, showing only 30 min intervals for visual clarity.

Significant shifts in vibrational modes were observed only in the fingerprint region (no significant peak shifts in the aliphatic region), and peak center frequencies of key vibrational modes are reported in Tables S1 and S2. The line shape maturation behavior is uniform and reproducible across trials for five of the six liquid films, suggesting a similar maturation process and outcome, as shown in Figure 4. The exception to this is our most asymmetric ionic liquid, [C_1_C_11_im]­[NTf_2_]. Despite being the most asymmetric IL in this study and having a reproducible maturation time of 540 ± 10 min, the spectral profile for [C_1_C_11_im]­[NTf_2_] at maturation is significantly different between 1000 cm^–1^ to 1400 cm^–1^ across several trials. Results from three independent replicate measurements are shown in Figure 5. The differences in the spectral profile suggest that these films’ final states are different, likely quasi-stable states with different molecular orientations. Work by Burankova et al. suggests that, while ILs with longer alkyl chains have slower global dynamics, the localized motions of the alkyl chains become faster as *n* increases.^46^ This is because the aliphatic tails exist in a larger nonpolar “pocket”, allowing more conformational freedom. The more dynamic alkyl tail rearrangement may lead to more cation/anion conformations; hence, the liquid films of these most asymmetric systems are more isotropic than the other IL systems studied here.

**Figure 4:**
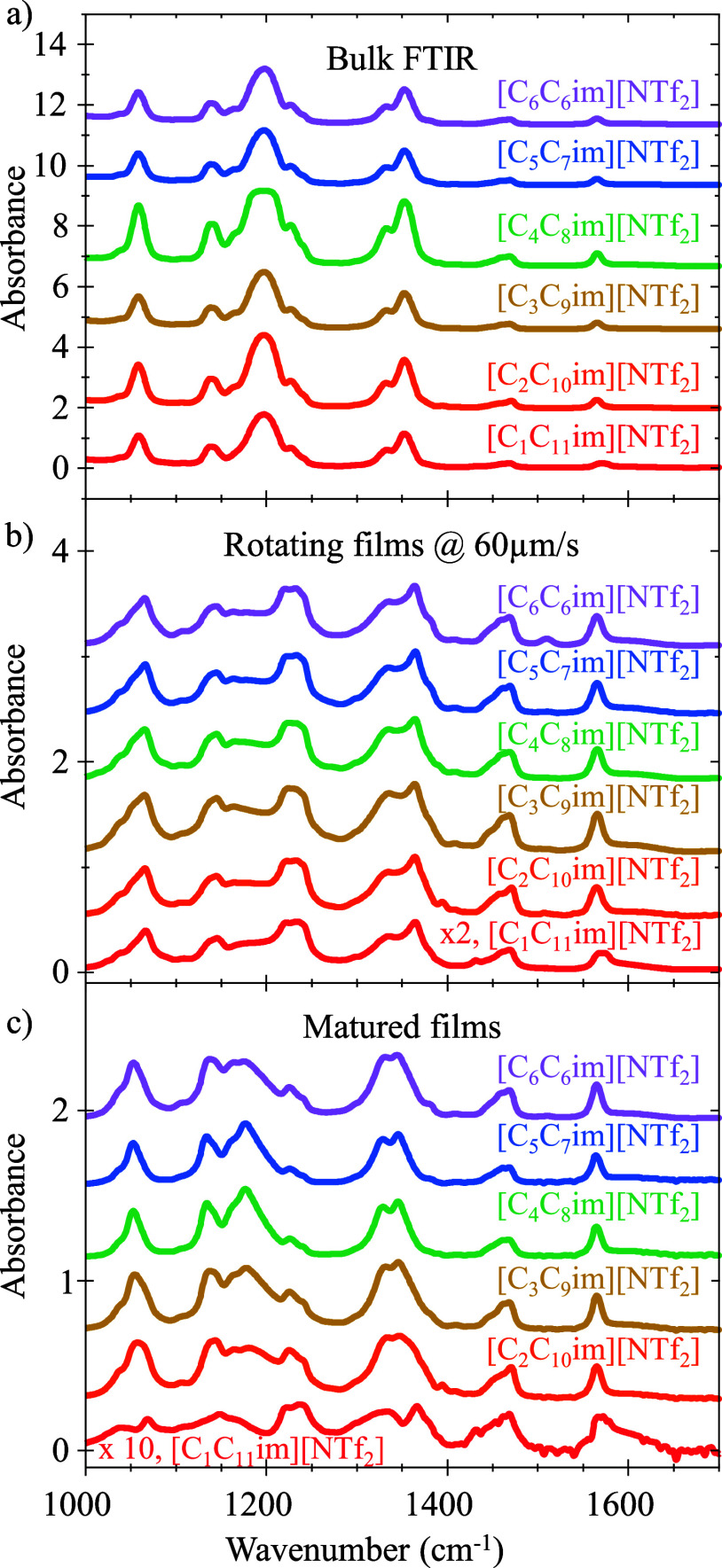
Representative FTIR spectra of bulk ILs (a) and IRRAS spectra of isotropic thin films on a rotating silver substrate at 60 μm/s (b) and anisotropic matured films (c) of ILs in this study, showing the changes in the spectral profile under different environments, yet similar profiles between ILs in the same environment. Spectra are offset for visual clarity. Film thicknesses are listed in Table 2, and a more detailed spectral analysis can be found in Figure S5.

**Figure 5:**
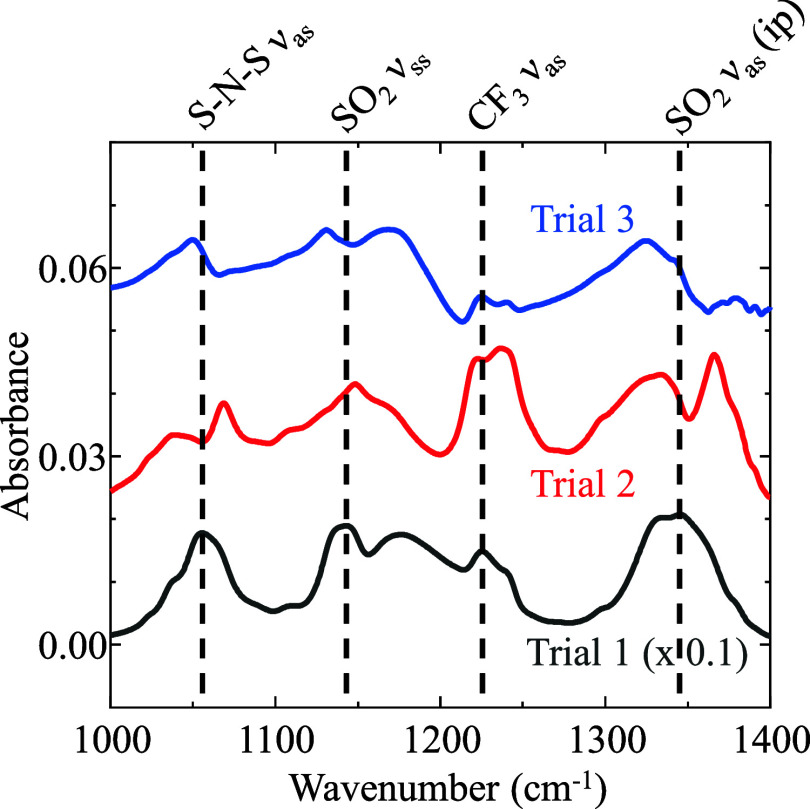
IRRAS spectra showing the spectral differences of three individually matured [C_1_C_11_im]­[NTf_2_] films. Spectra are offset for visual clarity.

Film thicknesses at maturation time points were determined using ellipsometry, as the film thickness at the time the film matures reveals the extent of ordering within the film. These maturation thicknesses or ordering lengths are listed in Table 2. The data in Table 2 show inconsistent relationships between film thickness and the time required to mature, meaning the extent of ordering is independent of the time it takes for the film to order. We note that film thickness for a mature [C_2_C_10_im]­[NTf_2_] film could not be determined as some peak center frequencies were still shifting (however slightly) at 10 h in each of the three replicate measurements. Therefore, Table 2 reports the film thickness of [C_2_C_10_im]­[NTf_2_] at 600 min. The extended maturation time for [C_2_C_10_im]­[NTf_2_] is most likely due to the combined effects of its higher viscosity and lower anion diffusion coefficient (Table 1) compared with the other 5 ILs, thus causing a slower shear rate. Ultimately, the ILs in this study displayed longer maturation times compared to ILs in previous studies with similar viscosities and molecular structures.^20^ This is attributed to the longer alkyl chains on the cations, which contribute to slow dynamics in IL systems.^47–50^

Figure S5 shows a comparison of infrared spectra for each [C_n_C_12‑n_im]­[NTf_2_] IL, revealing additional details of spectral profile changes as the films mature. The top (black) spectrum corresponds to the bulk transmission FTIR spectrum, the middle spectra (red) are the IRRAS spectra of a thin film on a silver substrate rotating at 60 μm/s, and the bottom (blue) is an IRRAS spectrum of the film once it reaches maturation. Across all ILs in this study, the most significant shift in vibrational mode center frequencies occurred in peaks associated with S–N–S ν_as_ (∼1059 cm^–1^), SO_2_ ν_ss_ (∼1140 cm^–1^), CF_3_ ν_as_ (∼1180 cm^–1^), SO_2_ ν_as_(ip) (∼1333 cm^–1^), and SO_2_ ν_as_(op) (∼1354 cm^–1^), as detailed in Table S1 and Figures S6 and S7. These shifts to lower energies during maturation are characteristic of the molecules shifting from an isotropic to an anisotropic orientation and the formation of ordered structures.^21,30^ Peaks associated with CF_3_ ν_as_ at ∼1225 cm^–1^ did not show a significant shift in center frequency but exhibited a significant decrease in absorbance (Figure S8), suggesting that its net dipole orientation is increasingly parallel to the substate in mature films. In all, the reorientation process can consist of translational or rotational movement or conformational changes within the molecule or a combination thereof. Thus, a more detailed analysis of these spectral changes can indicate how the ions are reorienting during the ordering process.

The [NTf_2_]^−^ anion is a flexible molecule that can easily rotate about the S–N–S bond interconverting between its *cis* (C_1_) and *trans* (C_2_) conformations.^51,52^ Shifts in certain [NTf_2_]^−^ vibrational modes can be an indicator of *cis* or *trans* conformations.^53–55^ The main rotamer marker is the S–N–S bending or wagging vibrational modes between 600 and 660 cm^–1^; however, this was outside our experimental window. Another notable marker within our experimental window is the SO_2_ ν_as_ “doublet” consisting of the ν_as_(ip) and ν_as_(op) modes. Both modes are present in both rotamers, though the ν_as_(ip) is known to be more intense for the *trans* rotamer than the *cis* rotamer and vice versa for the ν_as_(op).^53^ Therefore, by analyzing the SO_2_ ν_as_(ip) to SO_2_ ν_as_(op) peak absorbance ratio, we can qualitatively determine conformational and translational changes in [NTf_2_]^−^ as the films mature.

The *trans* rotamer is known to be more prevalent in bulk solutions at room temperature as it is more stable than the *cis* rotamer by ∼3 kJ/mol due to reduced steric hindrance of the CF_3_ groups and electron repulsion of the SO_2_ groups.^53–55^ This is reflected in our bulk FTIR results, Figure S5, with a ν_as_(ip) to ν_as_(op) ratio of 1.86 ± 0.03 to 1.92 ± 0.02 increasing with n for all ILs in this study, as listed in Table S3. In fresh films, this ratio reduces to between 1.1 ± 0.2 and 1.5 ± 0.3 (Table S3), which indicates an increase in *cis* rotamers in the film compared to bulk. During the maturation process, as seen in Figure S9, a continued spectral presence of both SO_2_ ν_as_ modes indicates that the SO_2_ dipoles maintain an orientation more perpendicular to the substrate; thus, the orientation of the [NTf_2_]^−^ anion translates into a parallel position to the surface. At maturation, the SO_2_ ν_as_ ratios for all IL films in this study were between 1.00 ± 0.04 to 1.13 ± 0.07, where [C_1_C_11_im]­[NTf_2_], [C_3_C_9_im]­[NTf_2_], and [C_4_C_8_im]­[NTf_2_] matured films have SO_2_ ν_as_ ratios within the standard deviation of their respective fresh film and [C_2_C_10_im]­[NTf_2_], [C_5_C_7_im]­[NTf_2_], and [C_6_C_6_im]­[NTf_2_] films have lower SO_2_ ν_as_ ratios at maturation than in their respective fresh film, as listed in Table S3. These results suggest an increased quantity of *cis* conformers in mature films compared with bulk or fresh films. Moreover, the matured film SO_2_ ν_as_ ratios of all ILs in this study were within error of each other, suggesting a more uniform ratio of *trans* to *cis* rotamers independent of the alkyl chain length.

Additionally, within the first two hours of the maturation process, in four of the ILs, the SO_2_ ν_as_ ratio significantly increases and then decreases again before plateauing prior to maturation occurring, Figure S9. This suggests significant interconversion of *cis* anions to *trans* back to *cis* as the molecules self-orient and the films mature. However, [C_1_C_11_im]­[NTf_2_] and [C_2_C_10_im]­[NTf_2_] films exhibited little change in the SO_2_ ν_as_ ratio during the maturation process. The SO_2_ ν_as_ ratio for [C_1_C_11_im]­[NTf_2_] stayed relatively constant over time, having a ratio of 1.3 ± 0.2 in fresh films and 1.1 ± 0.1 at maturation. This lack of conformational rotation of [NTf_2_]^−^ during maturation could be partly the reason why [C_1_C_11_im]­[NTf_2_] matures into different quasi-stable states. As for [C_2_C_10_im]­[NTf_2_], while no significant changes in the SO_2_ ν_as_ ratio were observed within the first two hours, the ratio in the mature film (1.00 ± 0.04) was lower than that in the fresh film (1.5 ± 0.3). We suggest that this slow conformational rotation is due to the aforementioned anion self-diffusion coefficient that is not only the slowest of the ILs in this study, but [C_2_C_10_im]­[NTf_2_] is also the only IL in this study whose anion diffusion coefficient is lower than its cation diffusion coefficient. This could also contribute to why the film did not mature within 10 h. These results suggest that the ability of [NTf_2_]^−^ to rotate is necessary for the film to completely mature.

Overall, increasing the cation asymmetry does increase the extent of ordering in the film within the IL film from 0.4 ± 0.2 μm for [C_6_C_6_im]­[NTf_2_] to ∼ 1.1 ± 0.9 μm for [C_2_C_10_im]­[NTf_2_]. [C_1_C_11_im]­[NTf_2_] shows an unexpected trend deviation again with a slightly lower maturation thickness of 0.7 ± 0.2 μm as shown in Figure 6. Previous studies by Anaredy et al. showed a strong linear correlation between maturation time and viscosity (*R* ^2^ = 0.9591) for various [NTf_2_]^−^ ILs.^20^ Conversely, in this study, IL isomers show no linear trend between the maturation time and viscosity, as shown in Figure S10a. Rather, our results show a linear correlation of *R* ^2^ = 0.8589 between the maturation thickness and viscosity, Figure S10b. The higher standard deviation for [C_2_C_10_im]­[NTf_2_] compared to other IL matured film thicknesses is due to the higher standard deviation from the ellipsometry measurements as seen in Figure 2.

**Figure 6:**
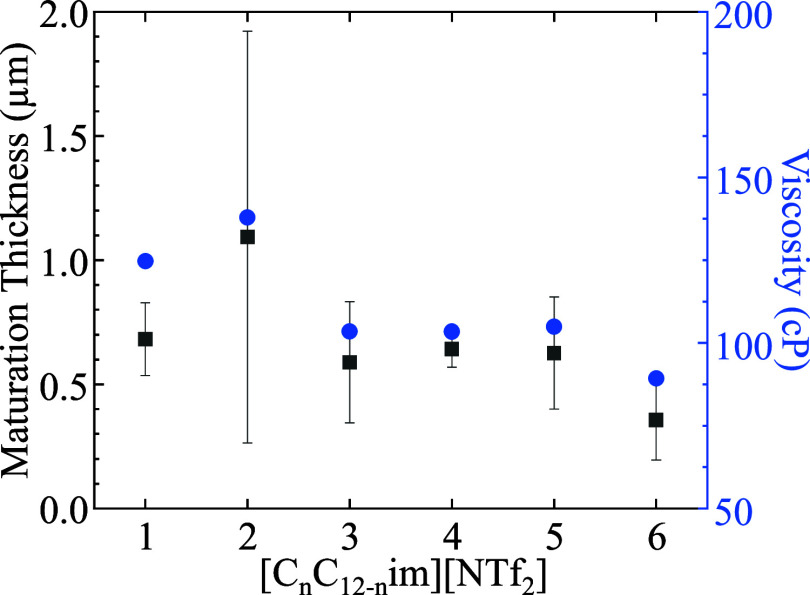
Film thicknesses of matured films (black) and bulk viscosity (blue) of the ionic liquids in this study as a function of symmetry. Error bars denote the pooled standard deviation (black) and standard deviation (blue) and might be smaller than the associated data point.

Studies on alkyl-based self-assembled monolayers and ionic liquids have shown that the alkyl chain extension (*cis* vs *gauche*) can be determined by comparing the peak absorbance ratio of the CH_2_ ν_ss_ and CH_3_ ν_ss_ modes.^56,57^ Tillman et al. show that as the alkyl chains orient more perpendicular to a surface, the CH_2_ ν_ss_ modes will decrease in absorbance, thus decreasing at this ratio.^56^ Harris et al. used this principle to show an increase in chain coupling and conformational ordering of alkyl chains in [P_666,14_]­[BH_4_] with decreasing temperature.^57^ We applied this principle here as a metric to reveal any subtle changes in the aliphatic stretching regions of our data. Such interactions could suggest interactions between adjacent alkyl tails, which would contribute to the formation of ordered structures within the film. The peak absorbance ratios of CH_2_ ν_ss_ (∼2860 cm^–1^) and CH_3_ ν_ss_ (∼2873 cm^–1^) were calculated for all spectra in each data set and are plotted in Figure 7 as a function of time since ceasing rotation. While most of the aliphatic mode peak frequencies exhibited minor shifting (less than the resolution of our measurement), we noted that the CH_3_ FR mode (∼2930 cm^–1^) in some individual trials exhibited shifts of up to 7 cm^–1^. These shifts returned to the initial frequencies at or prior to maturation (see the data in Figure S11). The changes in the CH_2_ ν_ss_/CH_3_ ν_ss_ ratio over time were reproducible regardless of the number of replicates containing shifts in the CH_3_ FR vibrational mode, as demonstrated by the error bars for five of the six ILs in Figure 7. The CH_2_ ν_ss_/CH_3_ ν_ss_ ratio did increase to some degree for all ILs prior to maturation, with [C_4_C_8_im]­[NTf_2_] having the greatest increase in ratio from an initial average ratio of 1.26 ± 0.08 to the highest average CH_2_ ν_ss_/CH_3_ ν_ss_ ratio, which was 1.51 ± 0.02 at maturation. This suggests that the alkyl tails of the ILs have a more parallel orientation to the surface at maturation in comparison to fresh films. Interestingly, [C_1_C_11_im]­[NTf_2_] shows high variability in the CH_2_ ν_ss_/CH_3_ ν_ss_ ratio, further suggesting different pathways from the bulk film to ordered structures, containing various numbers of gauche defects.

**Figure 7:**
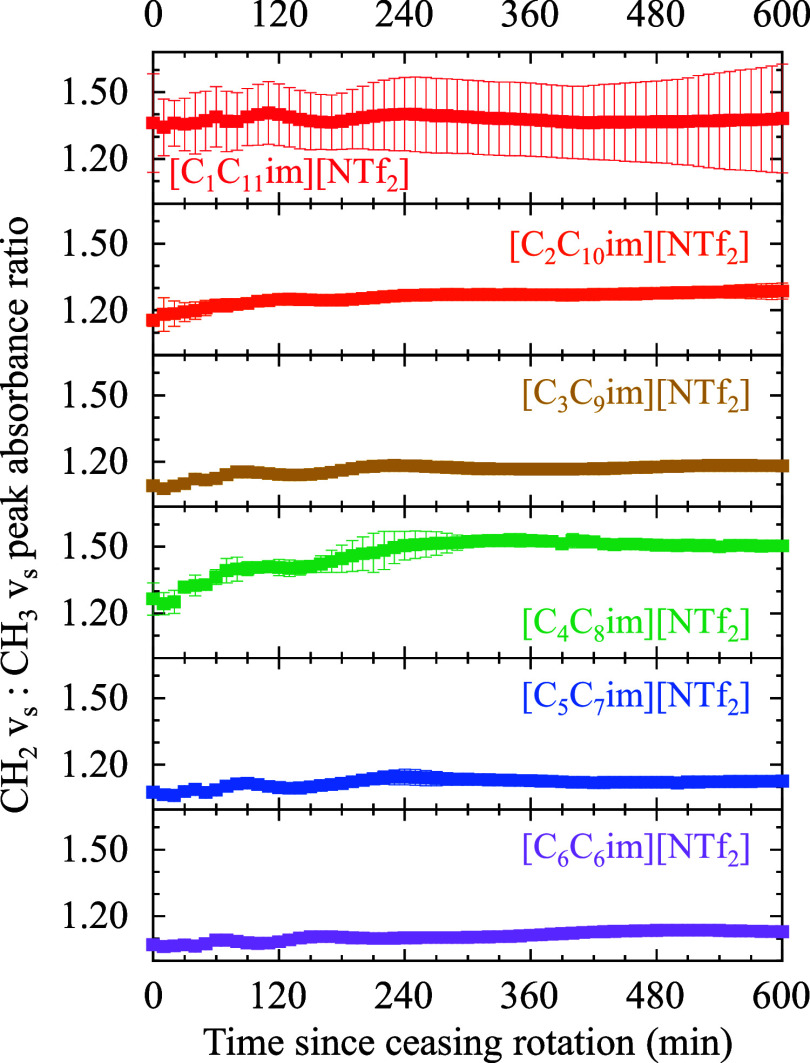
Peak absorbance ratios of the CH_2_ symmetric stretch at ∼2860 cm^–1^ vs the CH_3_ symmetric stretch at ∼2873 cm^–1^. Error bars denote the standard deviation of *n* ≥ 3.

To see if any connections could be drawn to the thermal phase transitions of these materials, we recorded DSC traces for each IL, as shown in Figure S12; tabulated data are shown in Table S4. Five of the six IL samples exhibited only a glass transition upon both cooling and heating. The exception to this is again [C_1_C_11_im]­[NTf_2_], which underwent a degree of crystallization upon cooling; then, upon heating, it underwent a glass transition, a series of overlapping exothermic cold crystallization, and/or solid–solid transitions, followed by a melting transition. Further studies are needed to fully define the transitions within the [C_1_C_11_im]­[NTf_2_]’s crystallization pathway, as previously published works have reported that the symmetry and alkyl chain length affect IL phase transition pathways. The work by Zheng et al. comparing symmetric 1,3-dialkylimidazolium ([C_*n*/2_C_*n*/2_im]­[NTf_2_], *n* = 4, 6, 8, and 10) and asymmetric 1-alkyl-3-methylimidazolium ([C_*n*–1_C_1_im]­[NTf_2_], *n* = 4, 6, 8, and 10) ILs showed that symmetric ILs with shorter alkyl chains (*n* = 4, 6) crystallize, while all asymmetric ILs and symmetric ILs with longer chains (*n* = 8, 10) only exhibit a glass transition.^35^ Specifically, small-wide-angle X-ray scattering data revealed a linear relationship between correlation length and alkyl chain length, showing greater apolar domain segregation and size in ILs with longer carbon chains. This suggests longer chains disrupt the packing efficiency and formation of an ordered lattice, especially when paired with a bulky anion with delocalized charge such as [NTf_2_]^−^. The work by Moschovi et al. and Abdurrokhman et al. studying [HC_*n*_im]­[NTf_2_] ILs (*n* = 0–12) and the work by Markiewicz et al. on alkyltriethylammonium bis­(trifluoromethylsulfonyl)­imide ([N_*n*222_]­[NTf_2_], *n* = 4–16) ILs report that only very short- and very long-alkyl-chain ILs undergo liquid–solid transitions, whereas intermediate-chain-length ILs only undergo glass transitions.^48,58,59^ Where the shorter *n* > 3 chains are primarily influenced by Coulombic forces, the longer decyl and dodecyl chains increase the separation between polar charged nanodomains, leading to better packing, thus increasing partial crystallinity. These works conclude that shifting the balance between structural ordering (e.g., nanodomains and intermolecular forces) and molecular flexibility (e.g., conformational entropy) leads to the observed changes in thermal behavior. Therefore, we suggest that [C_1_C_11_im]­[NTf_2_] has a more complex thermal phase transition pathway compared to its isomers due to longer alkyl chains having an increased number of possible configurations.

## Conclusion

This study investigated the effect of cation symmetry on the long-range ordering reported within IL films by analyzing the maturation thickness and infrared line shapes of six isomeric ILs. Time-resolved IRRAS spectra of IL films as they mature display shifts in center frequencies of vibrational modes toward lower energy, which suggests a more ordered system for all [C_n_C_12‑n_im]­[NTf_2_] IL films in this study. Our results show that ILs with more asymmetric cations lead to greater long-range ordering, except for [C_1_C_11_im]­[NTf_2_], which contains the longest aliphatic tail in our series and displays varied final IR profiles in its matured state, indicating varied molecular ordering in its matured state. Our results also showed that the [NTf_2_]^−^ anion’s ability to interconvert between rotamers is necessary for the films to completely mature. We also observed a minor odd–even alkyl chain length effect on the film thinning rate, which warrants further investigation using sum frequency generation or other surface-sensitive techniques to better understand the effect the odd vs even alkyl tail lengths and conformations have on thinning rates. These results add considerable information to our understanding of what drives interfacial long-range ordering, which is valuable in the design of various IL applications. In combination with previous works, these results show that both anion asymmetry and cation asymmetry play a major role in increasing the extent of long-range ordering.

## Supplementary Material



## Notes

Figure S1: ^1^H NMR of the six “zipper” ILs in this study before spectroscopic experimentation and after recovery as described in the Experimental Section; Figure S2: density and viscosity measurements for the six ILs in this study as a function of temperature; Figure S3: film thicknesses of the ionic liquid in this study measured by spectroscopic ellipsometry over time after ceasing rotation of 60 μm/s showing the development of a slight odd/even chain length effect between 50 and 150 min after ceasing rotation; Figure S4 representative IRRAS spectra of a [C_4_C_8_im]­[NTf_2_] thin film on a stationary silver substrate after rotating 60 μm/s at 1 h intervals showing the changes in vibrational modes as the film matures; Table S1: key low-frequency vibrational mode assignments for [C_*n*_C_12–*n*_im]­[NTf_2_] in various conditions; Table S2: aliphatic vibrational mode assignments for [C_*n*_C_12–*n*_im]­[NTf_2_] in various conditions; Figure S5: representative FTIR spectra of bulk and IRRAS spectra of thin films on a rotating silver substrate at 60 μm/s and matured films of ionic liquids in this study; Figure S6: center frequencies and associated peak absorbances for the S–N–S ν_as_ vibrational mode; Figure S7: center frequencies and associated peak absorbances for SO_2_ ν_ss_ and ν_as_(op) vibrational modes; Figure S8: center frequencies and associated peak absorbances for the CF_3_ ν_as_ vibrational mode; Table S3: center frequency peak absorbance ratios of the SO_2_ ν_as_(ip) to SO_2_ ν_as_(op) vibrational modes of [C_*n*_C_12–*n*_im]­[NTf_2_] under different environments; Figure S9: center frequency peak absorbance ratios of the SO_2_ ν_as_(ip) to SO_2_ ν_as_(op) vibrational modes as films mature over time of three or more individually matured films; Figure S10: maturation time and maturation thickness versus viscosity for [C_*n*_C_12–*n*_im]­[NTf_2_] (*n* = 1–6); Figure S11: center frequencies for the CH_3_ FR vibrational mode for three or more individually matured films; Figure S12: DSC thermograms of fresh [C_*n*_C_12–*n*_im]­[NTf_2_] (*n* = 1–6), cooled, and then heated with a heat flux of 5 °C/min showing glass transitions, exothermic crystallization, and endothermic melting transitions; and Table S4: phase transition temperatures and associated enthalpies of [C_*n*_C_12–*n*_im]­[NTf_2_] with 95% CI determined via differential scanning calorimetry, *n* ≥ 3 (PDF)
