# Supplementary material for: Effect of Cation Symmetry on the Long-Range Ordering in Ionic Liquid Films
Source: Langmuir. 2025 Nov 14;42(2):1905–15. doi: 10.1021/acs.langmuir.5c03333 (PMC12825391; doi:10.1021/acs.langmuir.5c03333)
Supplement: Supplementary file 1 [file la5c03333_si_001.pdf]

Supporting information for:

Effect of cation symmetry on the long-range ordering in ionic liquid films

Colleen B. Lasar [1] – colleen-lasar@uiowa.edu
Andrew Horvath [1] – andrew-horvath@uiowa.edu
Michael B. Van Den Top [1] – michael-vandentop@uiowa.edu
Spyridon Koutsoukos [2] – s.koutsoukos18@imperial.ac.uk
Tom Welton [2]* – t.welton@imperial.ac.uk
Scott K. Shaw [1]* – scott-k-shaw@uiowa.edu

[1] Department of Chemistry, The University of Iowa, Iowa City, IA 52242, United States
[2] Department of Chemistry, Imperial College, London SW7 2AZ, United Kingdom

Figure S1. ^{1}H NMR of the six 'zipper' ILs in this study before spectroscopic experimentation and after recovery as described in the experimental section. p S2

Figure S2. Density and viscosity measurements for the six ILs in this study as a function of temperature. p S3

Figure S3. Film thicknesses of the ionic liquid in this study measured by spectroscopic ellipsometry over time after ceasing rotation of 60 µm/s showing the development of a slight odd/even chain length effect between 50 – 150 mins after ceasing rotation. p S4

Figure S4. Representative IRRAS spectra of a [C_4C_8im][NTf_2] thin film on a stationary silver substrate after rotating 60 µm/s at one-hour intervals showing the changes in vibrational modes as the film matures. p S5

Table S1. Key low frequency vibrational mode assignments for [C_nC_{12-n}im][NTf_2] in various conditions. p S6

Table S2. Aliphatic vibrational mode assignments for [C_nC_{12-n}im][NTf_2] in various conditions. p S7

Figure S5. Representative FTIR spectra of bulk and IRRAS spectra of thin films on a rotating silver substrate at 60 µm/s and matured films of ionic liquids in this study. p S8

Figure S6. Center frequencies and associated peak absorbances for S-N-S ν_{as} vibrational mode. p S9

Figure S7. Center frequencies and associated peak absorbances for SO_2 ν_{ss} and ν_{as}(op) vibrational modes. p S10

Figure S8. Center frequencies and associated peak absorbances for CF_3 ν_{as} vibrational mode. p S11

Table S3. Center frequency peak absorbance ratios of the SO_2 ν_{as}(ip) to SO_2 ν_{as}(op) vibrational modes of [C_nC_{12-n}im][NTf_2] under different environments. p S12

Figure S9. Center frequency peak absorbance ratios of the SO_2 ν_{as}(ip) to SO_2 ν_{as}(op) vibrational modes as films mature over time of three or more individually matured films. p S13

Figure S10. Maturation time and maturation thickness verses viscosity for [C_nC_{12-n}im][NTf_2] (n = 1 – 6). p S14

Figure S11. Center frequencies for CH_3 FR vibrational mode for three or more individually matured films. p S15

Figure S12. DSC thermograms of fresh [C_nC_{12-n}im][NTf_2] (n = 1 – 6) cooled then heated with a heat flux of 5 °C/min showing glass transitions, exothermic crystallization, and endothermic melting transitions. p S16

Table S4. Phase transition temperatures and associated enthalpies of [C_nC_{12-n}im][NTf_2] with 95% CI determined via differential scanning calorimetry, n ≥ 3. p S17

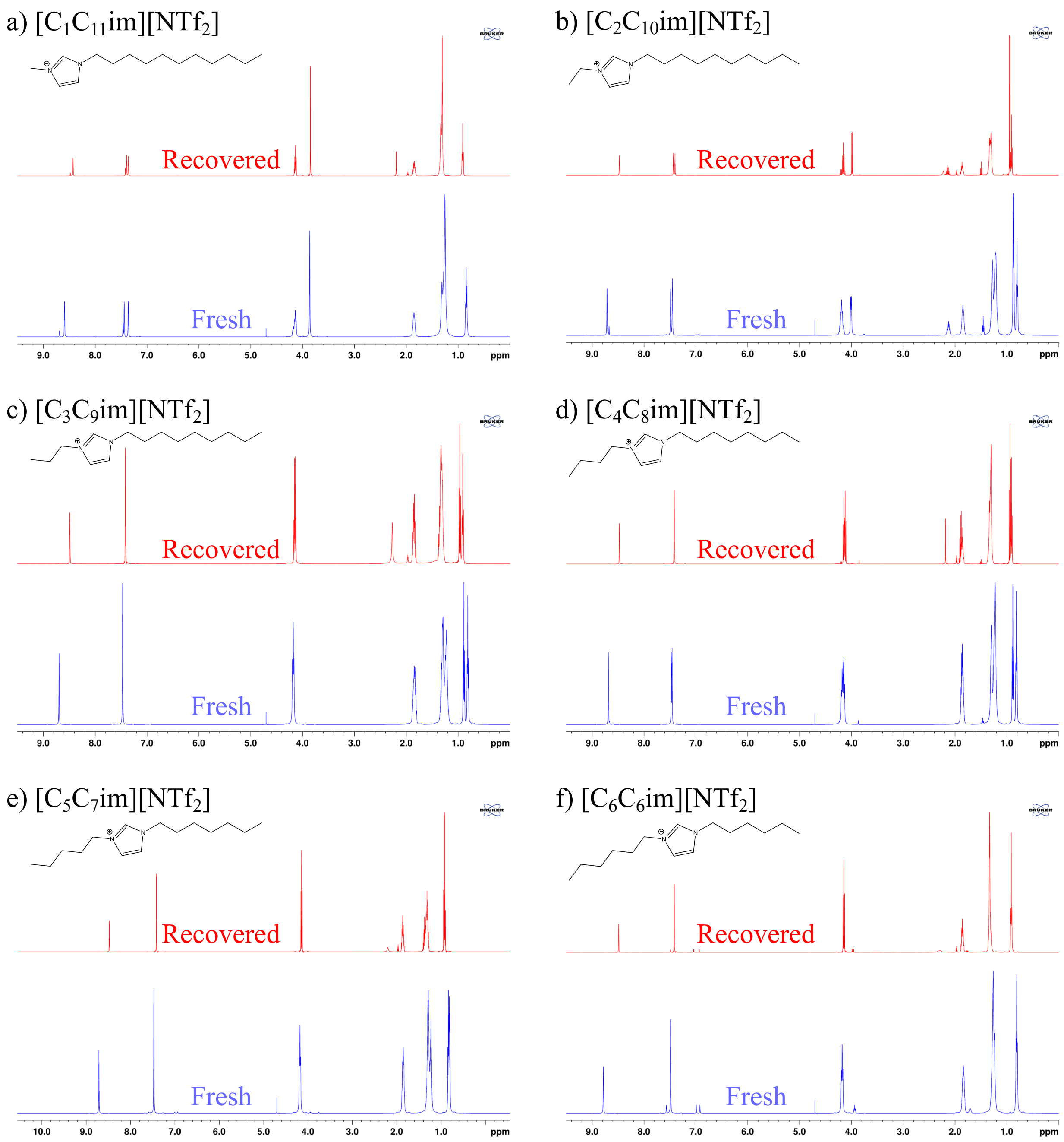

Figure S1. ^{1}H NMR of the six 'zipper' ILs in this study before spectroscopic experimentation (blue) and after recovery as described in the experimental section (red). Small changes in chemical shift and resolution are due difference in NMR sample preparation; fresh IL samples were prepared using a D_2O capillary and recovered IL samples were diluted in CD_3CN.

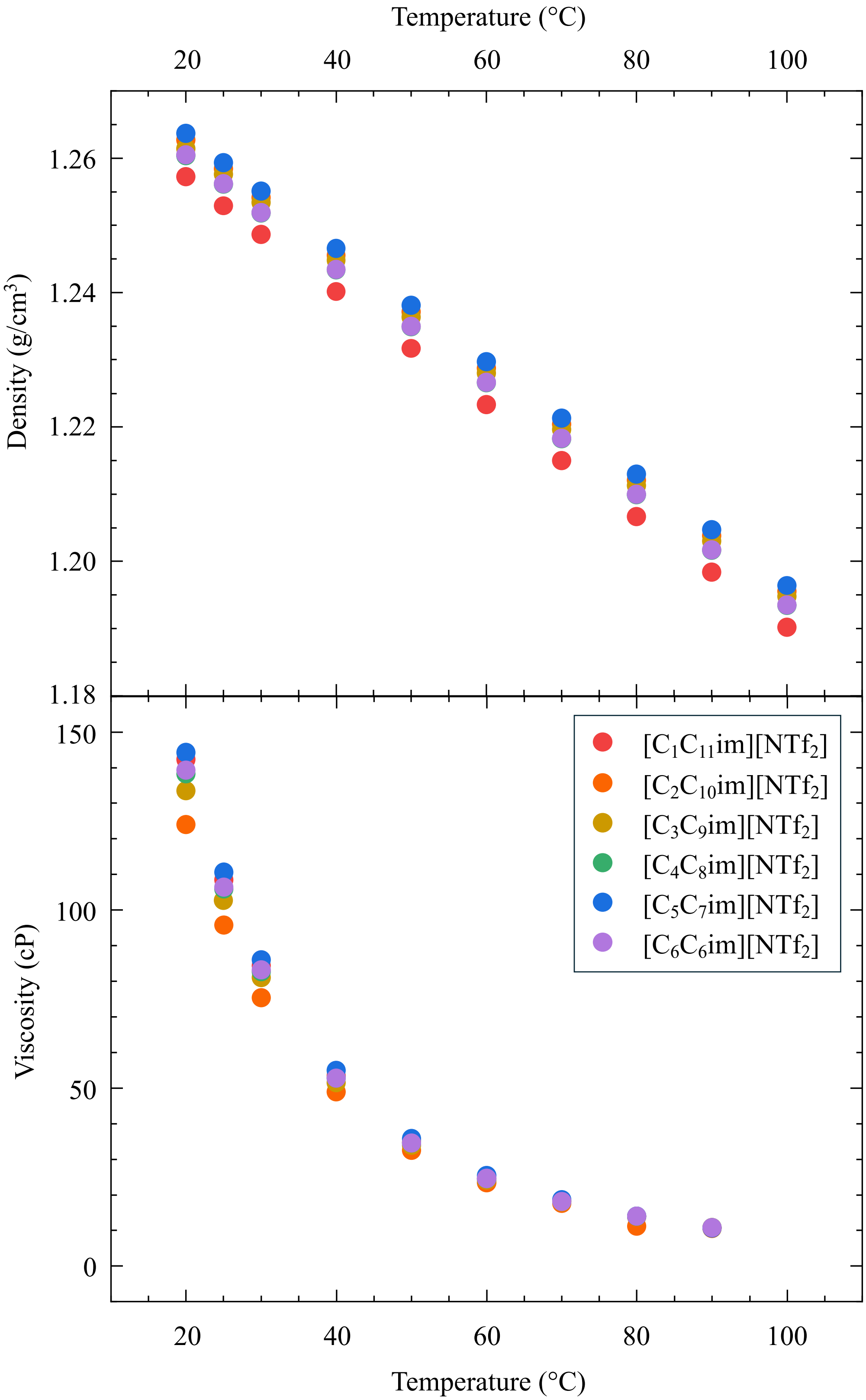

Figure S2. Density and viscosity measurements for the six ILs in this study as a function of temperature. Some data points are not visible due to overlapping data points.

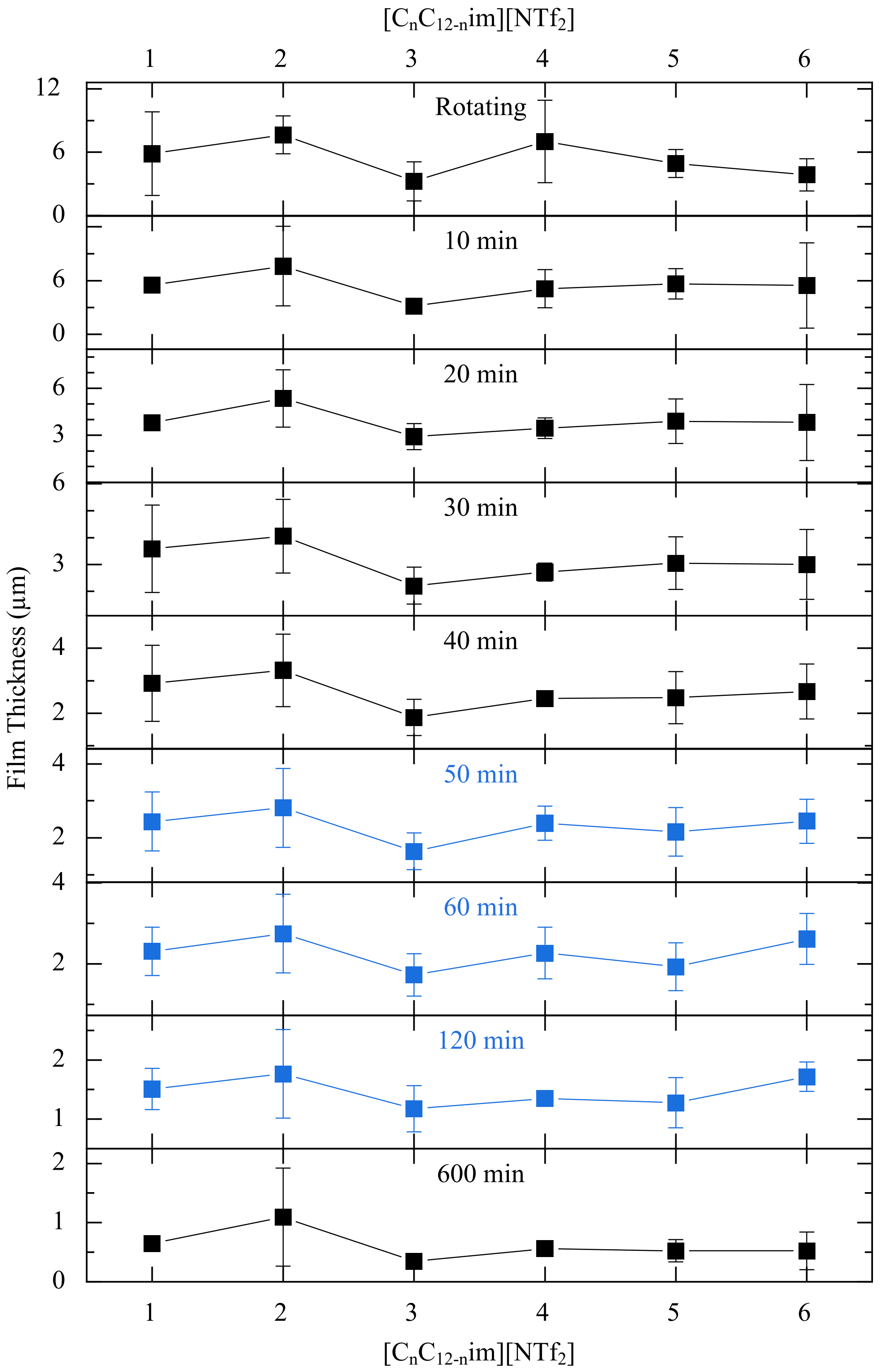

Figure S3. Film thicknesses of the ionic liquid in this study measured by spectroscopic ellipsometry over time after ceasing rotation of 60 μm/s showing the development of a slight odd/even chain length effect between 50 – 150 mins after ceasing rotation (blue). Error bars represent standard deviation of $n \geq 3$ independent trials and might be smaller than the data marker.

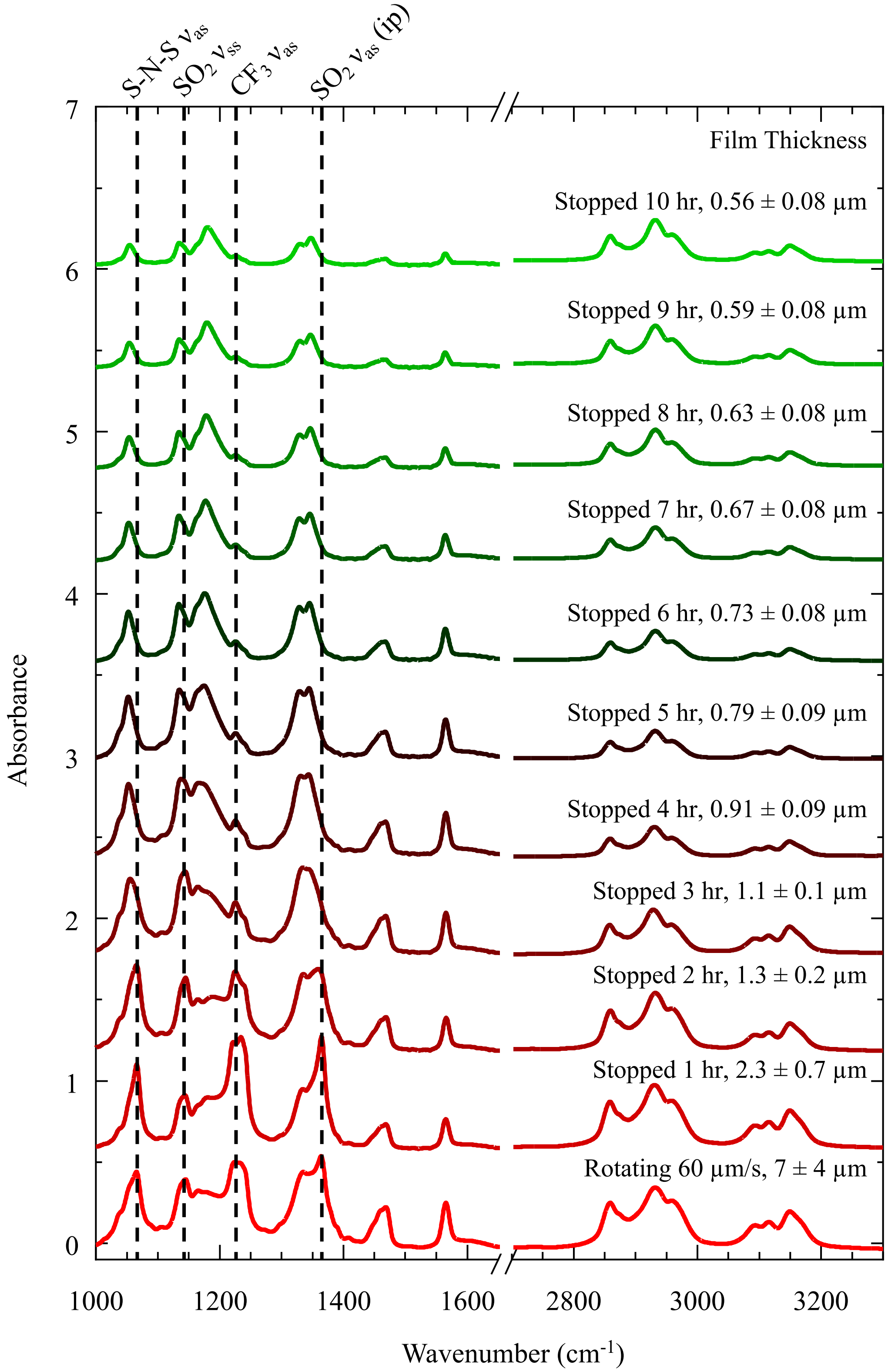

Figure S4. Representative IRRAS spectra of a [C_4C_8im][NTf_2] thin film on a stationary silver substrate after rotating 60 μm/s (bottom) at one-hour intervals showing the changes in vibrational modes as the film matures. Spectra are offset for visual clarity. Due to occasional changes in baseline as refractive indexes of the film changes as the film matures, a baseline subtraction step was added to the Find Peak method in Origin. This allowed more accurate monitoring of changes in peak absorbance and peak ratios as the films mature. Baseline points of 99.4443, 1665.7868, 1826.59898, 2104.13343, 2493.98115, 2635.53299, 3400.907, and 4000.118 cm^{-1}.

Table S1. Key low frequency vibrational mode assignments for $[C_nC_{12-n}im][NTf_2]$ in various conditions. All frequency values are in unites of cm^{-1} and those in red denote peaks with notable shifting in center frequency after maturation. Sh denotes shoulders.

	Vibrational Mode	S-N-S ν_{as}	SO_2 ν_{ss}	CF_3 ν_{as}	CF_3 ν_{as}		SO_2 ν_{as} (op)	SO_2 ν_{as} (ip)
$[C_1C_{11}im]$ $[NTf_2]$	**FTIR Bulk**	1059	1138	1197	1227	Sh:1242	1333	1352
	**IRRAS Rotating**	1063	1143	1175	1223	1235	1335	1365
	**IRRAS Matured**	1058	1141	1172	1224	Sh:1239	1331	1345
$[C_2C_{10}im]$ $[NTf_2]$	**FTIR Bulk**	1058	1138	1197	1227	Sh:1242	1332	1352
	**IRRAS Rotating**	1063	1145	1163	1223	1234	1335	1364
	**IRRAS Matured**	1056	1142	1181	1225	Sh:1240	1333	1345
$[C_3C_9im]$ $[NTf_2]$	**FTIR Bulk**	1058	1138	1197	1227	Sh:1242	1333	1352
	**IRRAS Rotating**	1062	1144	1164	1224	1234	1337	1364
	**IRRAS Matured**	1054	1136	1177	1225	Sh:1240	1331	1345
$[C_4C_8im]$ $[NTf_2]$	**FTIR Bulk**	1059	1140	1197	1227	Sh:1242	1333	1352
	**IRRAS Rotating**	1066	1145	1165	1223	1233	1335	1364
	**IRRAS Matured**	1053	1134	1177	1226	Sh:1240	1329	1345
$[C_5C_7im]$ $[NTf_2]$	**FTIR Bulk**	1058	1138	1198	1227	Sh:1242	1333	1352
	**IRRAS Rotating**	1063	1144	1163	1223	1233	1336	1364
	**IRRAS Matured**	1054	1135	1178	1226	Sh:1240	1330	1346
$[C_6C_6im]$ $[NTf_2]$	**FTIR Bulk**	1058	1138	1198	1227	Sh:1242	1333	1352
	**IRRAS Rotating**	1065	1145	1163	1221	1232	1336	1364
	**IRRAS Matured**	1053	1137	1175	1225	Sh:1240	1331	1344

Table S2. Aliphatic vibrational mode assignments for $[C_nC_{12-n}im][NTf_2]$ in various conditions. Shoulders are notated as Sh. Frequencies represent the range of averages for all six ILs in this study.

Vibrational Mode	FTIR Bulk, cm^{-1}	IRRAS Rotating, cm^{-1}	IRRAS Matured, cm^{-1}
CH_2 ν_{ss}	2858 – 2863	2858 – 2863	2857 – 2863
CH_3 ν_{ss}	2873	Sh: 2873 – 2874	Sh: 2873 – 2874
CH_3 FR / CH_2 ν_{as}	2928 – 2935	2932 – 2936	2929 – 2935
CH_3 ν_{as}	2957 – 2962	2958 – 2962	2957 – 2963
C(2)H ν	3092 – 3103	3091 – 3103	3091 – 3104
HC(4)C(5)H ν_{as}	3115 – 3121	3115 – 3120	3115 – 3120
HC(4)C(5)H ν_{ss}	3149 – 3156	3148 – 3155	3148 – 3155

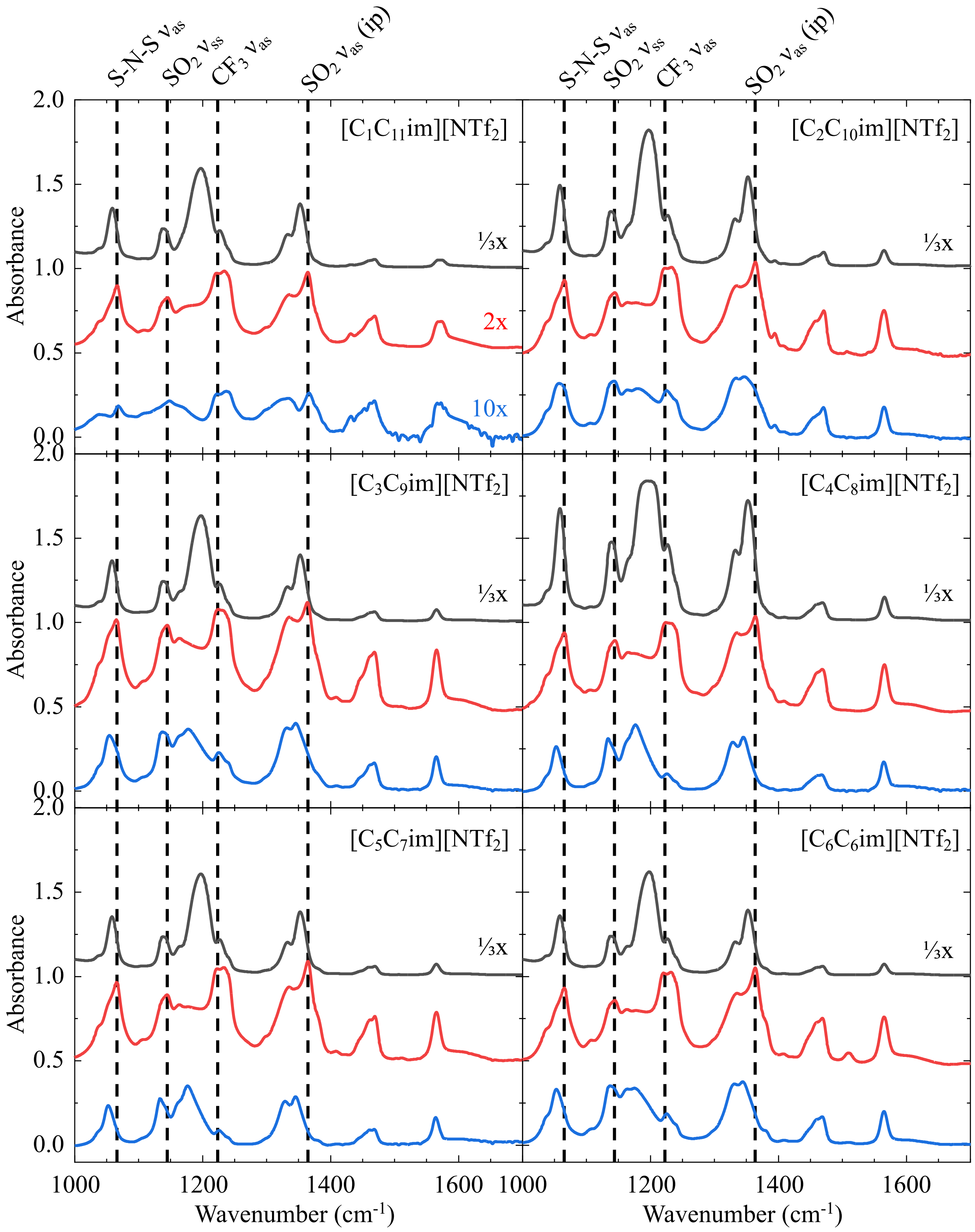

Figure S5. Representative FTIR spectra of bulk (black) and IRRAS spectra of thin films on a rotating silver substrate at 60 μm/s (red) and matured (blue) films of ionic liquids in this study. Vertical dashed lines indicate vibrational modes that lose intensity or shift frequency as the film matures. Bulk spectra are scaled by one third and spectra are offset for visual clarity.

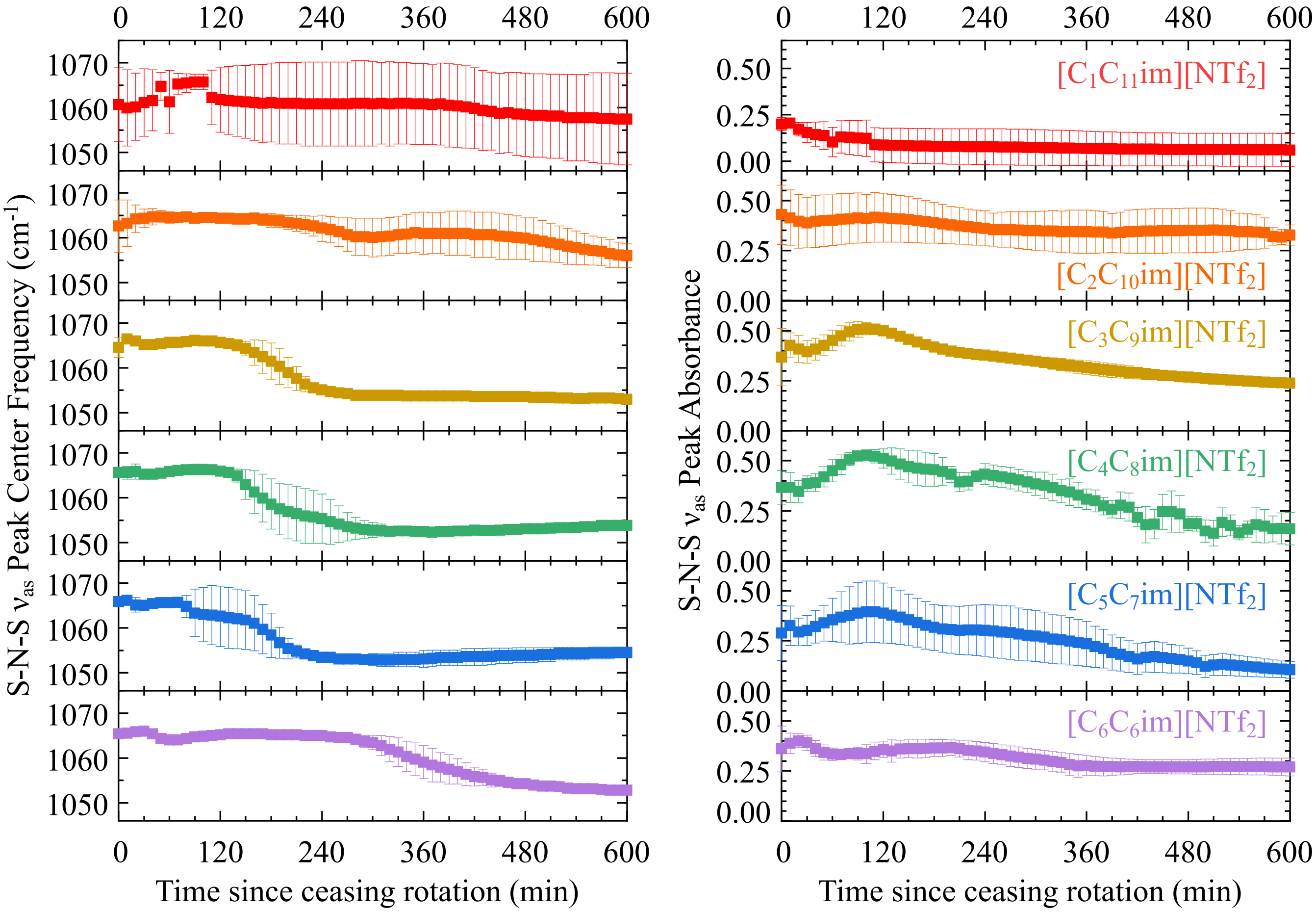

Figure S6. Center frequencies (left) and associated peak absorbances (right) for S-N-S asymmetric stretch vibrational mode. Error bars denote standard deviation of $n \geq 3$.

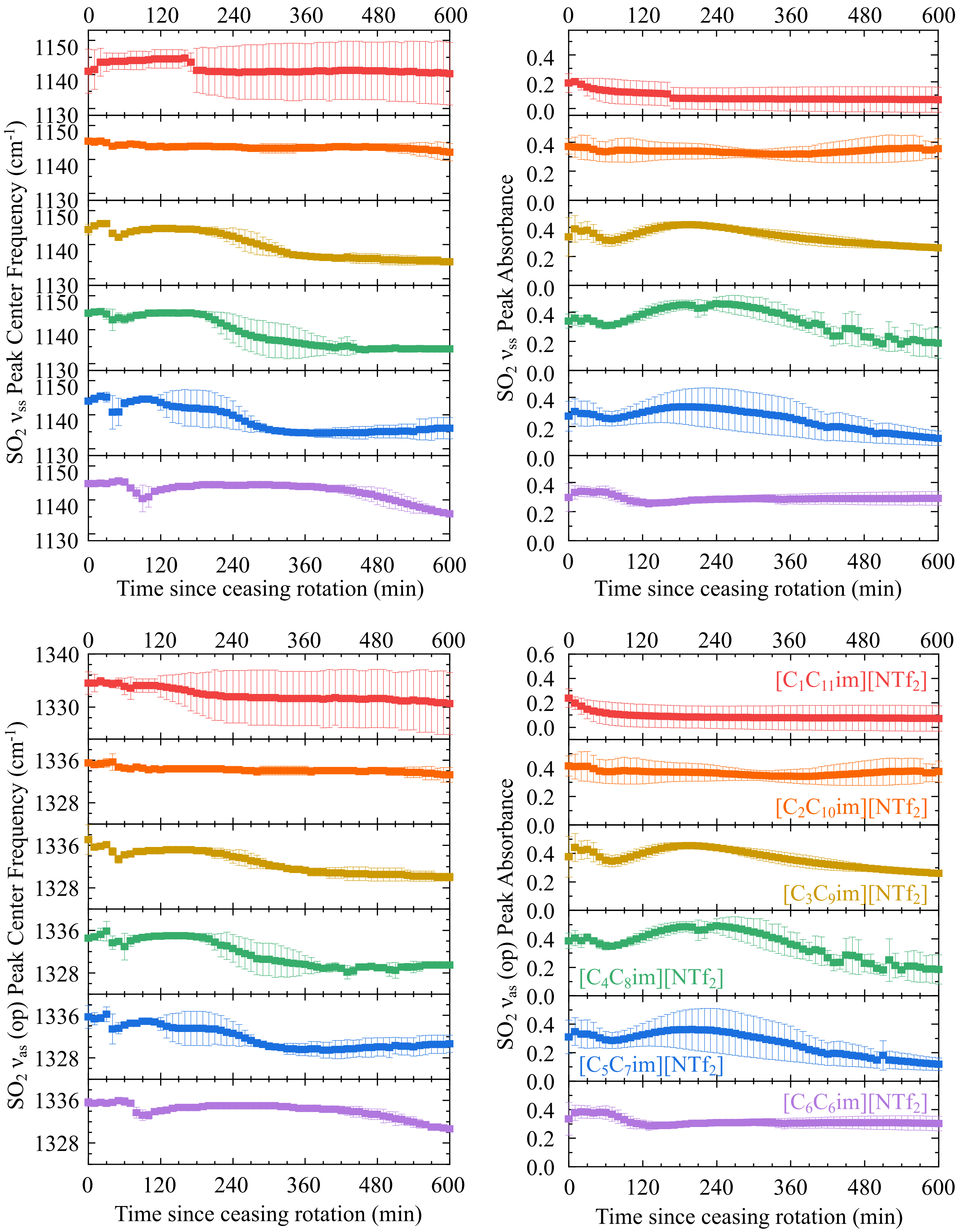

Figure S7. Center frequencies (left) and associated peak absorbances (right) for SO_2 symmetric (top) and asymmetric out-of-phase (bottom) stretch vibrational modes. Error bars denote standard deviation of $n \geq 3$.

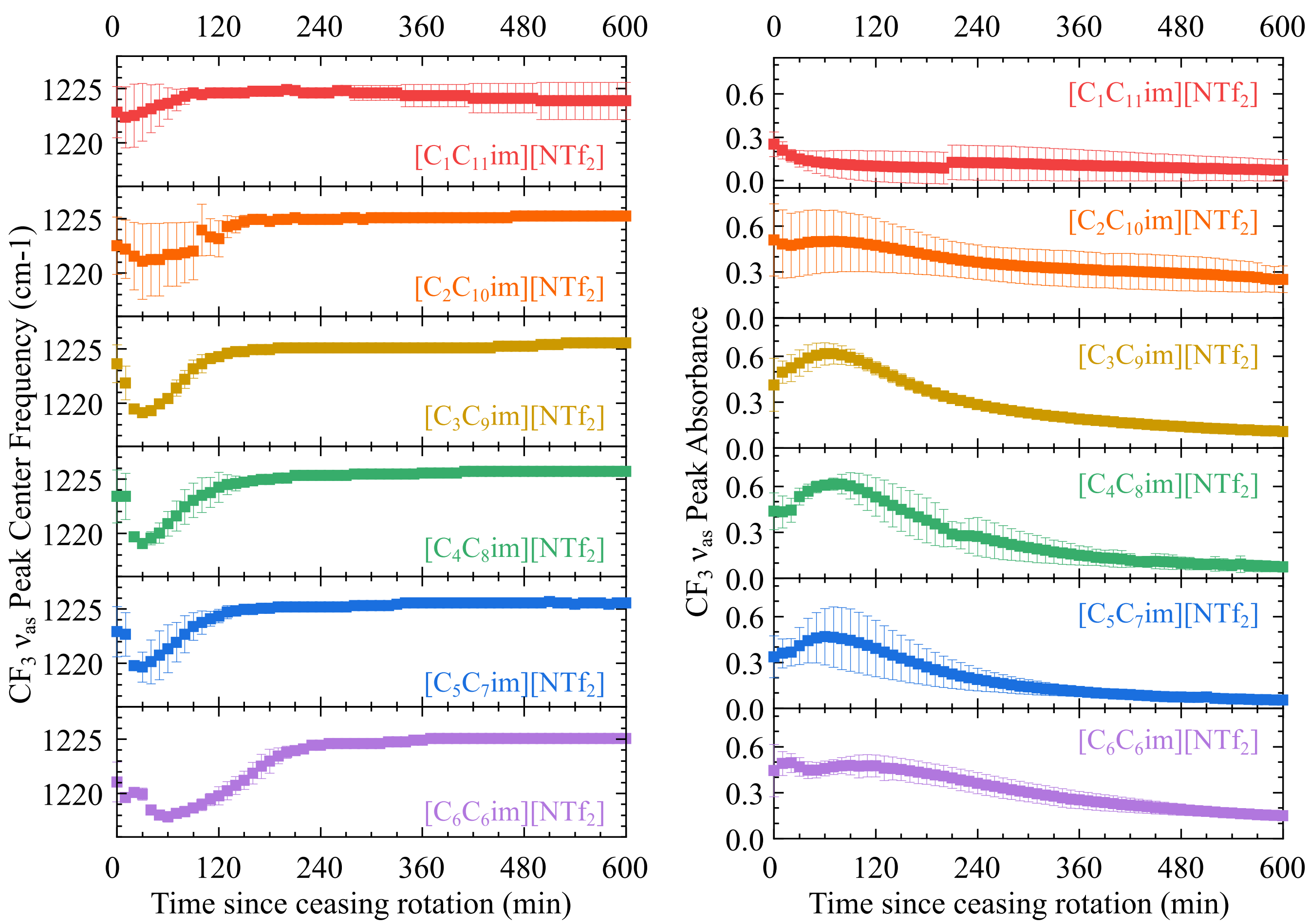

Figure S8. Center frequencies (left) and associated peak absorbances (right) for CF_3 asymmetric stretch vibrational mode. Error bars denote standard deviation of $n \geq 3$.

Table S3. Center frequency peak absorbance ratios of the SO_2 in-phase to out-of-phase asymmetric stretch vibrational modes of $[C_nC_{12-n}im][NTf_2]$ under different environments. Error denotes standard deviation of n ≥ 3 independent trails.

Ionic Liquid	FTIR Bulk	IRRAS Rotating	IRRAS Matured
$[C_1C_{11}im][NTf_2]$	1.86 ± 0.03	1.3 ± 0.2	1.1 ± 0.1
$[C_2C_{10}im][NTf_2]$	1.88 ± 0.02	1.5 ± 0.3	1.00 ± 0.04
$[C_3C_9im][NTf_2]$	1.90 ± 0.02	1.2 ± 0.3	1.06 ± 0.02
$[C_4C_8im][NTf_2]$	1.9 ± 0.1	1.1 ± 0.2	1.11 ± 0.08
$[C_5C_7im][NTf_2]$	1.90 ± 0.02	1.3 ± 0.1	1.13 ± 0.07
$[C_6C_6im][NTf_2]$	1.92 ± 0.02	1.4 ± 0.1	1.04 ± 0.2

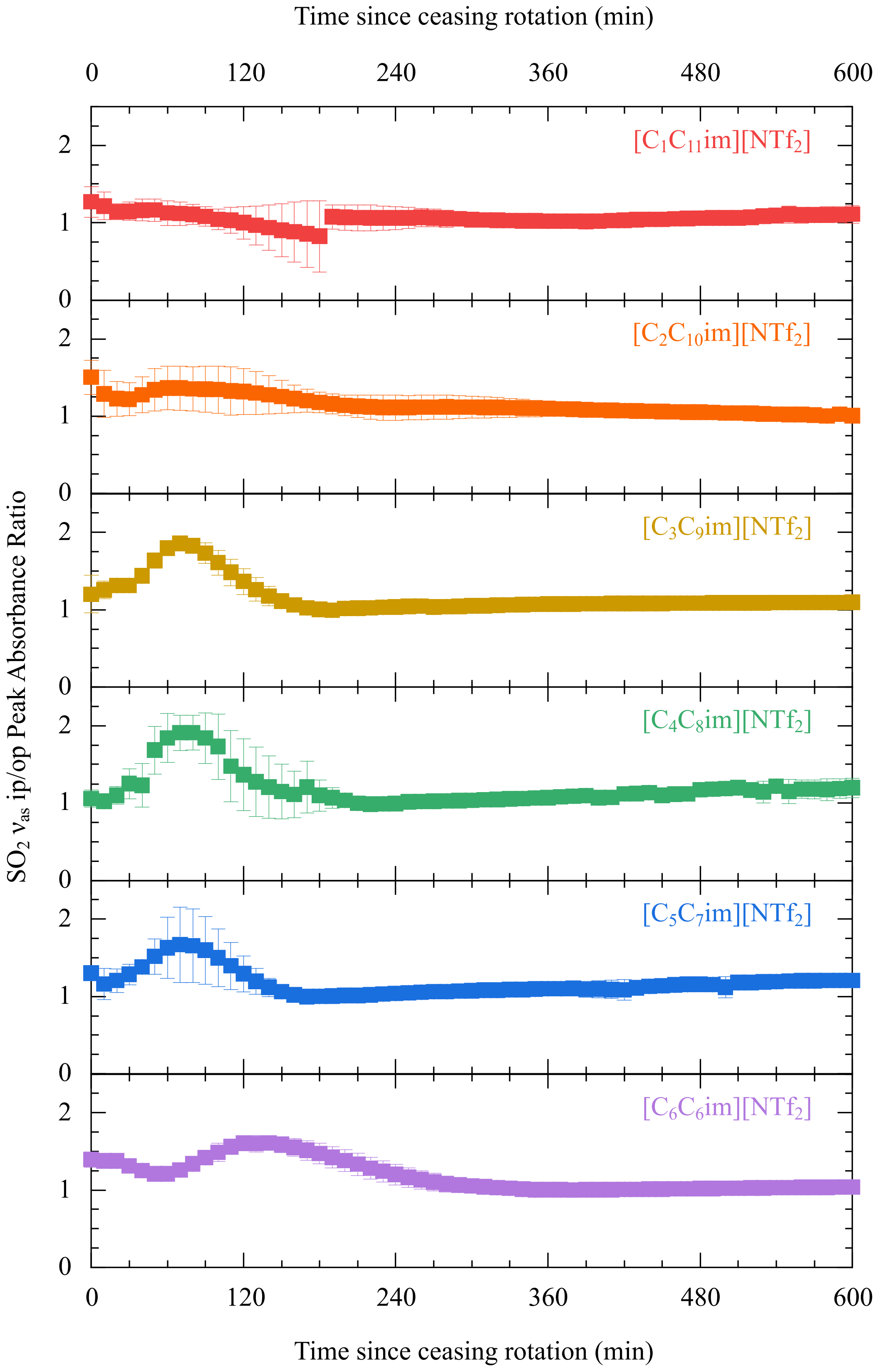

Figure S9. Center frequency peak absorbance ratios of the SO_2 in-phase to out-of-phase asymmetric stretch vibrational modes as films mature over time of three or more individually matured films.

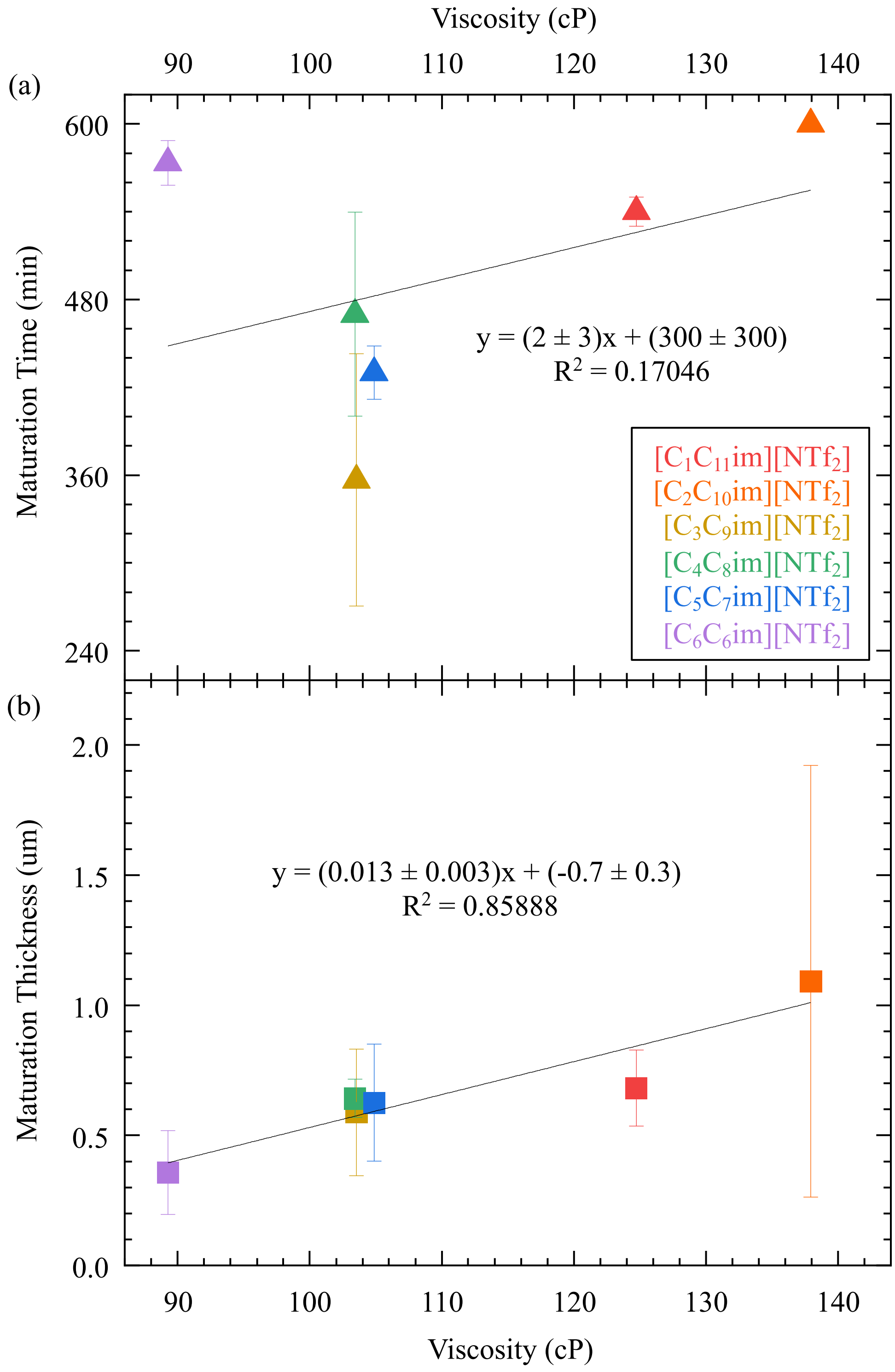

Figure S10. Maturation time (a) and maturation thickness (b) verses viscosity for [C_1C_{11}im][NTf_2] (red), [C_2C_{10}im][NTf_2] (orange), [C_3C_9im][NTf_2] (yellow), [C_4C_8im][NTf_2] (green), [C_5C_7im][NTf_2] (blue), and [C_6C_6im][NTf_2] (purple). Error bars show standard deviation. X-error bars may be smaller than data symbol.

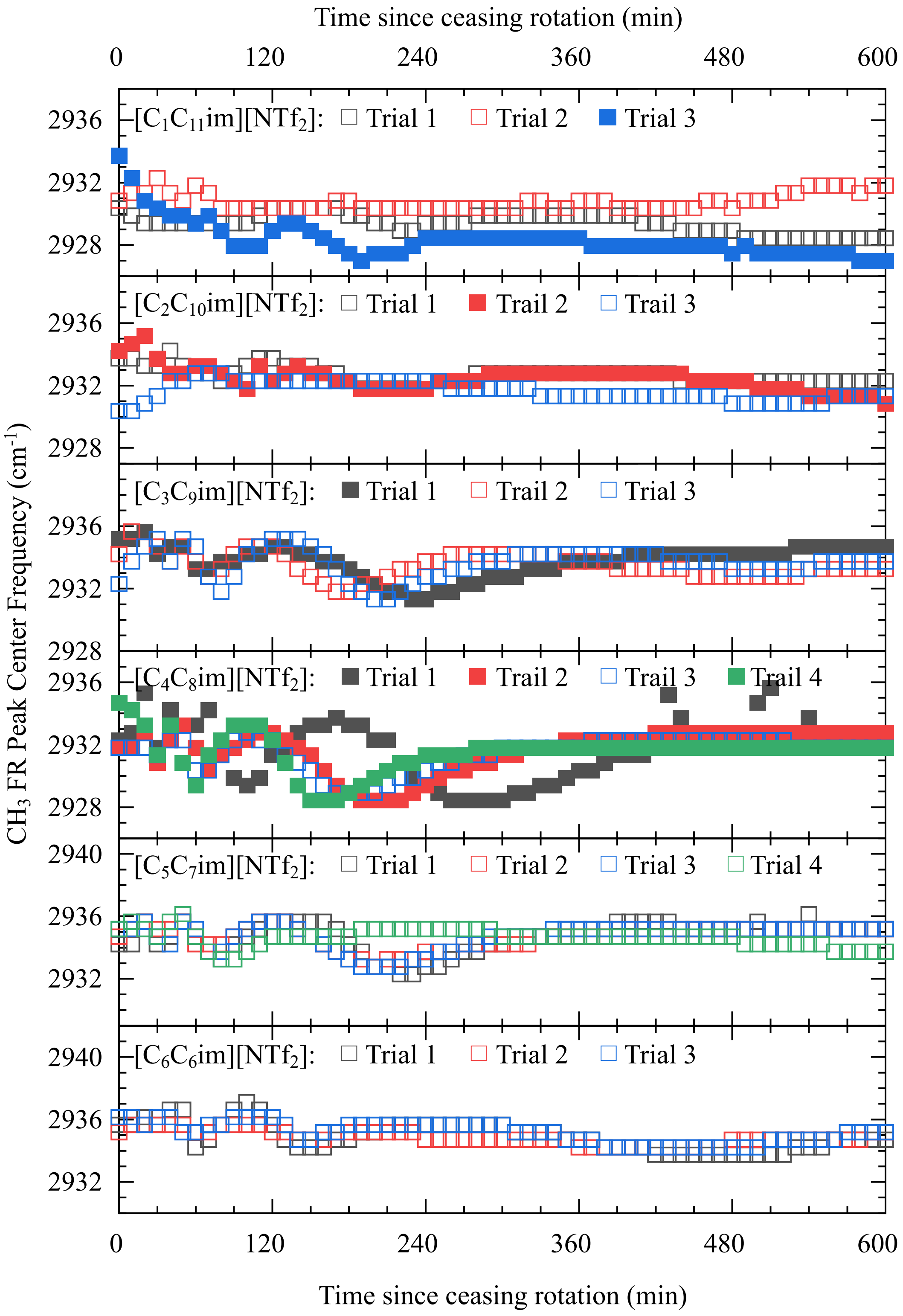

Figure S11. Center frequencies for CH_3 FR vibrational mode for three or more individually matured films. Solid and hollow data points denote trials where center frequency shifted greater than or less than 4 cm^{-1} respectively.

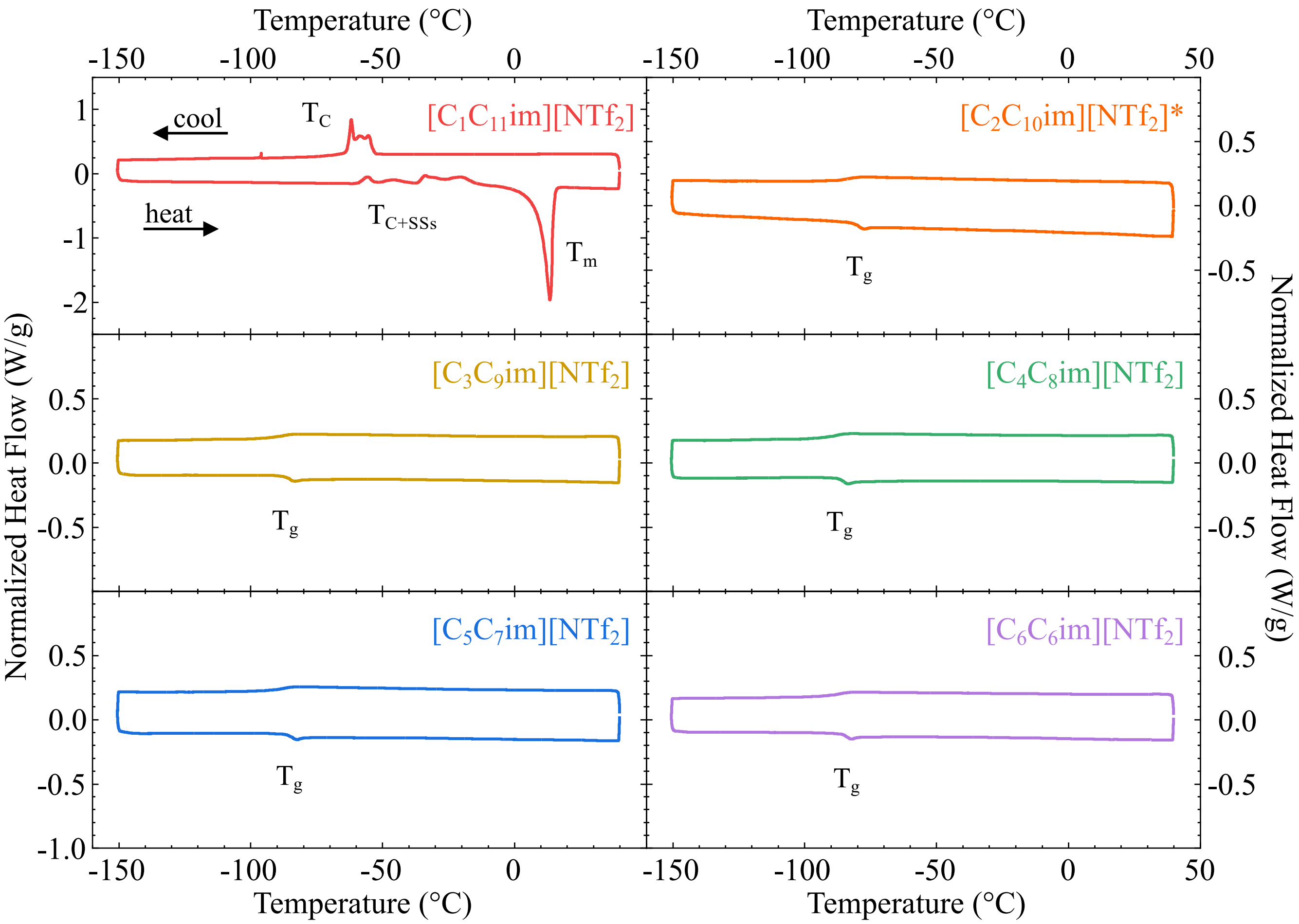

Figure S12. DSC thermograms of fresh $[C_nC_{12-n}im][NTf_2]$ where n = 1 – 6 cooled then heated with a heat flux of 5 °C/min showing glass transitions (T_g), an exothermic crystallization (T_{CC}), and an endothermic melting transition (T_m). The asterisk notes thermal trace of the recovered IL. Change in baseline displayed for $[C_2C_{10}im][NTf_2]$ is attributed to the calibration of the instrument as it was witnessed in both fresh and recovered ILs.

Table S4. Phase transition temperatures and associated enthalpies of $[C_nC_{12-n}im][NTf_2]$ with 95% CI determined via differential scanning calorimetry, n ≥ 3.

Ionic Liquid	Crystallization, T_C	Glass Transition, T_g	Series of Cold Crystallization & Solid-Solid, T_{C+SSs}	Melting, T_m
$[C_1C_{11}im][NTf_2]$	-57 ± 4 °C<br>37 ± 6 W/g	-83.8 ± 0.3 °C	-54 ± 3 °C<br>37 ± 8 W/g	-13.2 ± 0.3 °C<br>-96 ± 6 W/g
$[C_2C_{10}im][NTf_2]$	-	-80.5 ± 0.4 °C	-	-
$[C_3C_9im][NTf_2]$	-	-86.4 ± 0.2 °C	-	-
$[C_4C_8im][NTf_2]$	-	-86.8 ± 0.2 °C	-	-
$[C_5C_7im][NTf_2]$	-	-85.7 ± 0.3 °C	-	-
$[C_6C_6im][NTf_2]$	-	-85.2 ± 0.2 °C	-	-
